# Thermally reversible Diels–Alder linkers enable reusable methacrylate supports for enzyme immobilisation

**DOI:** 10.1039/d6cc02525k

**Published:** 2026-08-25

**Authors:** Emmanouil Broumidis, Francesca Paradisi

**Affiliations:** a Department of Chemistry, Biochemistry and Pharmaceutical Sciences, University of Bern Freiestrasse 3 CH-3012 Bern Switzerland francesca.paradisi@unibe.ch

## Abstract

Spent enzyme immobilisation resins constitute microplastic waste. Here, commercially available C_2_-amine methacrylate beads are converted into reusable supports through furan decoration and a thermally cleavable Diels–Alder linker bearing an NHS ester. The enzyme–linker assembly is removed by retro-Diels–Alder reaction, regenerating the support over multiple cycles.

## Abstract 2

Commercially available C_2_-amine methacrylate beads are converted into reusable enzyme supports through furan decoration and a thermally cleavable Diels–Alder linker, reducing microplastic waste from spent carriers.

## Graphical abstract

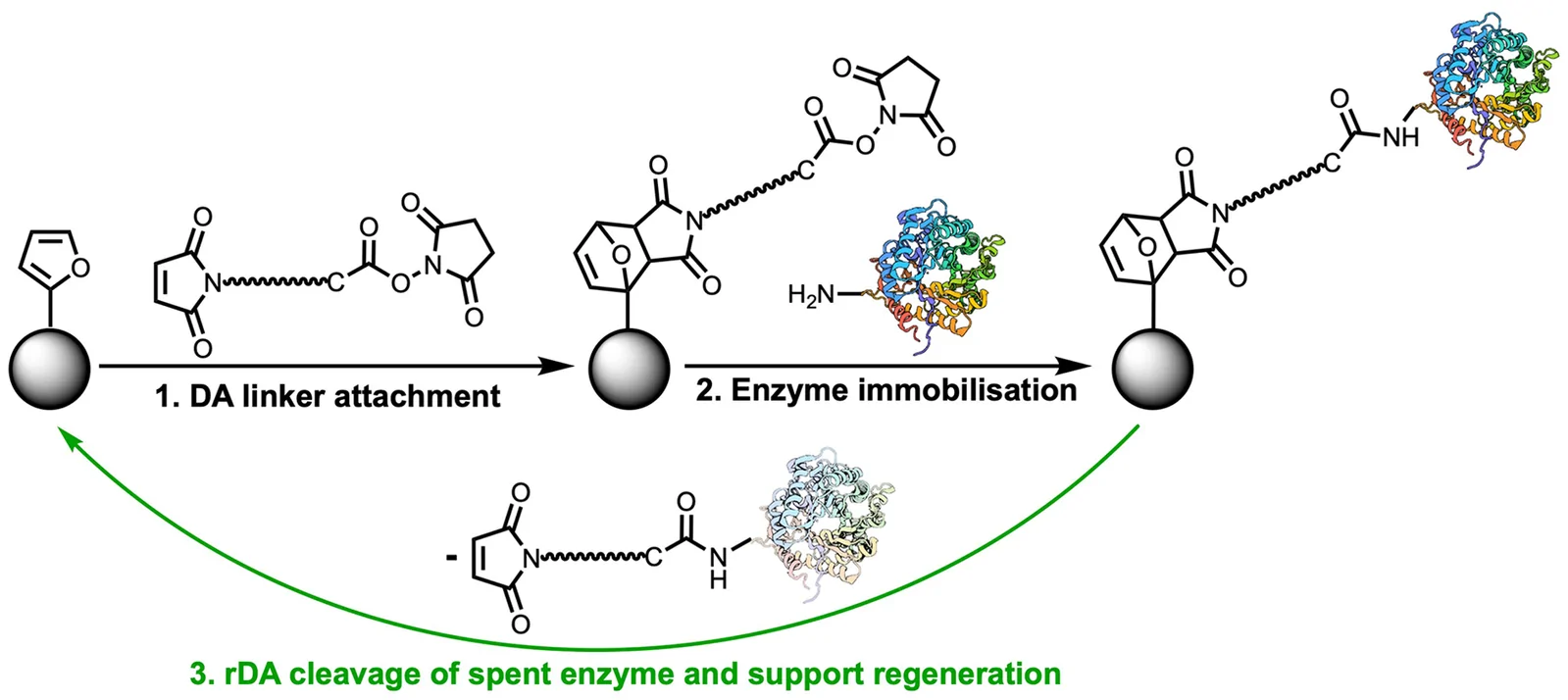

Enzyme immobilisation on solid supports is a cornerstone of industrial biocatalysis, enabling straightforward catalyst recovery, enhanced operational stability, and seamless integration into continuous processes.^1,2^ Methacrylate-based resins represent one of the most widely adopted carrier platforms for covalent immobilisation, with several commercial formulations (*e.g.* Purolite®, ReliZyme™) available bearing epoxy, amino, or other reactive functionalities.^3^ However, after the immobilised enzyme has lost its catalytic competence, the polymeric support, typically comprising >90% of the total catalyst mass, is discarded as waste.^4^ Given the microbead morphology of these carriers, this practice introduces non-degradable microplastics into the waste stream, raising growing environmental concerns (see SI, Fig. S1). Despite the maturity of immobilisation technology, strategies for regenerating and reusing the support itself remain remarkably scarce. Non-covalent alternatives such as physical adsorption, ionic exchange, and affinity capture (*e.g.* His-tag/Ni^2+^) are inherently reversible, but enzyme leaching and narrow operational windows often restrict their use in practice.^5–7^ Regenerating supports after covalent immobilisation, however, has proved far more challenging. Our group recently demonstrated the feasibility of support regeneration through reversible boronate ester chemistry.^4^ While DA reactions have been applied to biomolecule conjugation on surfaces,^8,9^ polymer particles,^10^ and in nanomedicine,^11^ their use for support regeneration in biocatalysis is, to the best of our knowledge, unprecedented. Herein, we report a complementary approach exploiting the well-established thermoreversibility of the Diels–Alder (DA) reaction to achieve the same goal using simple, widely available reagents.

Our strategy centres on the conversion of commercially available C_2_-amine methacrylate resin (Purolite®) into a furan-decorated support through reductive amination with furfural, a cheap and renewable feedstock.^12,13^ The pendant furan groups serve as dienes for a [4+2] cycloaddition with a commercially available bifunctional maleimide–containing linker, bearing a *N*-hydroxysuccinimide (NHS) terminus, covalently installing reactive NHS esters on the resin surface for enzyme conjugation *via* exposed lysine residues.^14^ Crucially, after the biocatalytic process is complete and the enzyme has been exhausted, the support can be regenerated by thermally inducing the retro-DA reaction, which cleaves the linker–enzyme assembly and restores the furan moieties for a fresh DA coupling cycle.

To validate the DA/retro(r) DA chemistry in a spectroscopically transparent system, we first employed soluble, non-crosslinked poly(vinylbenzyl chloride) (PVBC). Furan-2-ylmethanol was deprotonated with NaH and coupled to the pendant chloride groups.^15^ The resulting furanyl polymer was then reacted with the bifunctional MAL-PEG_2_-NHS linker *via* DA reaction (Scheme 1). NMR spectroscopy confirmed successful DA adduct formation and thermal reversibility was established (SI, Fig. S2–S4). Having established the DA/retro-DA cycle on this soluble polymer, we also probed whether it could double as an immobilisation matrix, taking advantage of the NHS esters now installed along its backbone. However, no binding was detected for any of the model proteins tested (a dehydrogenase, a glycosyl hydrolase, a fluorescent protein, and a serum albumin) (SI, page S8). We ascribe this failure to the hydrophobic, non-porous character of non-crosslinked PVBC, which restricts aqueous protein access to the surface NHS esters.^16^

**Scheme 1:**
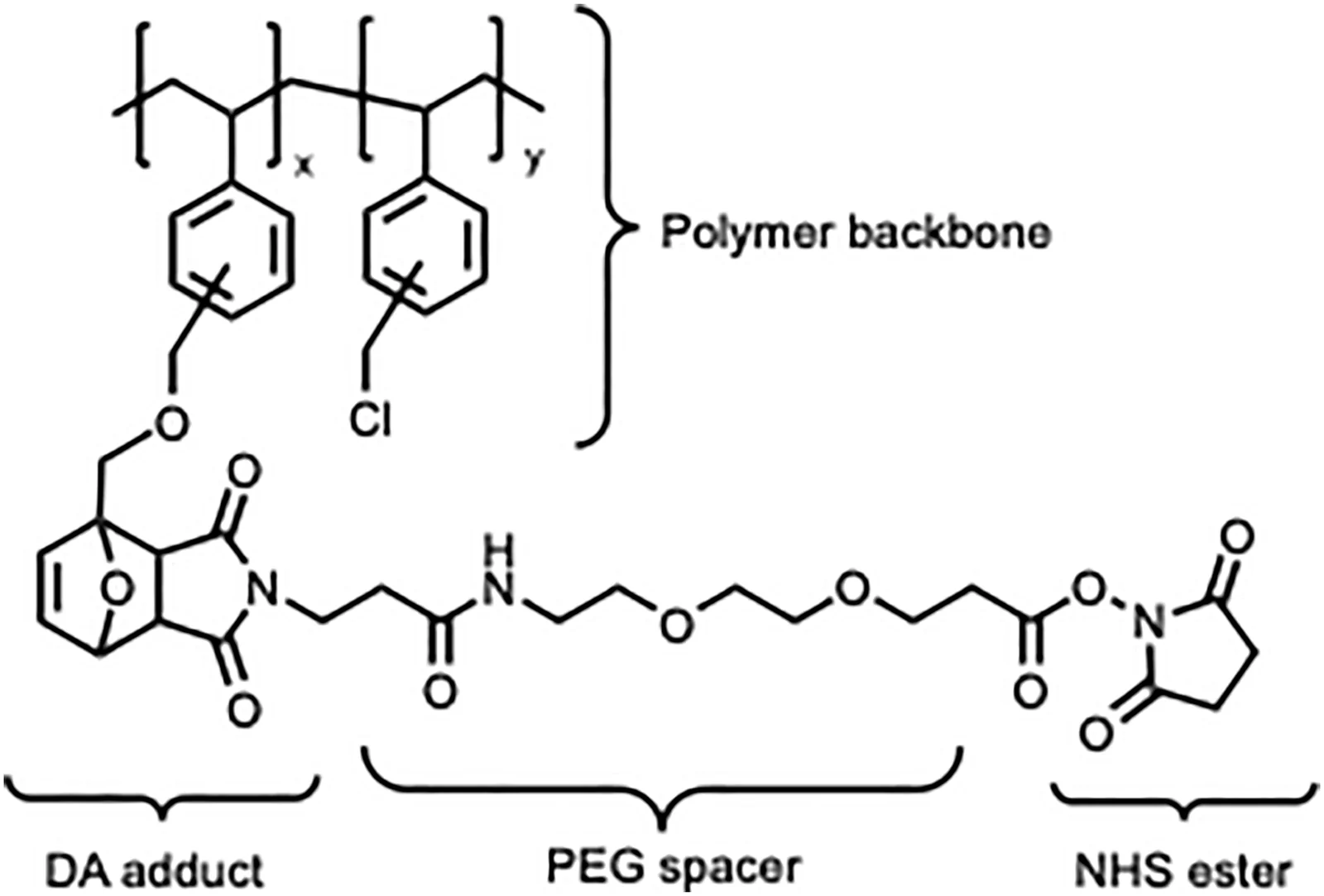
Structure of non-crosslinked PVBC based polymer used in this study.

We therefore decided to investigate this chemistry on the commercial, porous, hydrophilic crosslinked C_2_-amine methacrylate resin. Treatment with an excess of furfural (5 eq.) and sodium cyanoborohydride (NaBH_3_CN) in methanol for 4 h afforded the furan-decorated support (Fig. 1a and SI, page S9).^17^ Consumption of the primary amine was confirmed by the ninhydrin test (SI, Fig. S5) and FT-IR spectroscopy of the resulting material, showing the appearance of a characteristic furan C–O–C stretch at around 1065 cm^−1^ (SI, Fig. S6).^18^ DA reaction with the MAL-PEG_2_-NHS linker was then carried out in *N*,*N*-dimethylacetamide (DMAc) for 72 h, a timeframe previously found necessary for quantitative conversion with polymeric substrates using this linker (Fig. 1b).^19^ Thermal elemental analysis provided a convenient method for monitoring DA coupling (Fig. 1c): the three additional nitrogen atoms introduced by each linker molecule allowed calculation of the NHS loading from the incremental N wt% increase. After the first 72 h reaction, a loading of approximately 62 µmol of active NHS ester per gram of resin was determined, which falls within the range typically offered by commercial immobilisation supports with epoxy or amino functional handles (40–100 µmol g^−1^).^3,20^ Detailed calculations are provided in the SI, Fig. S7.

**Fig. 1:**
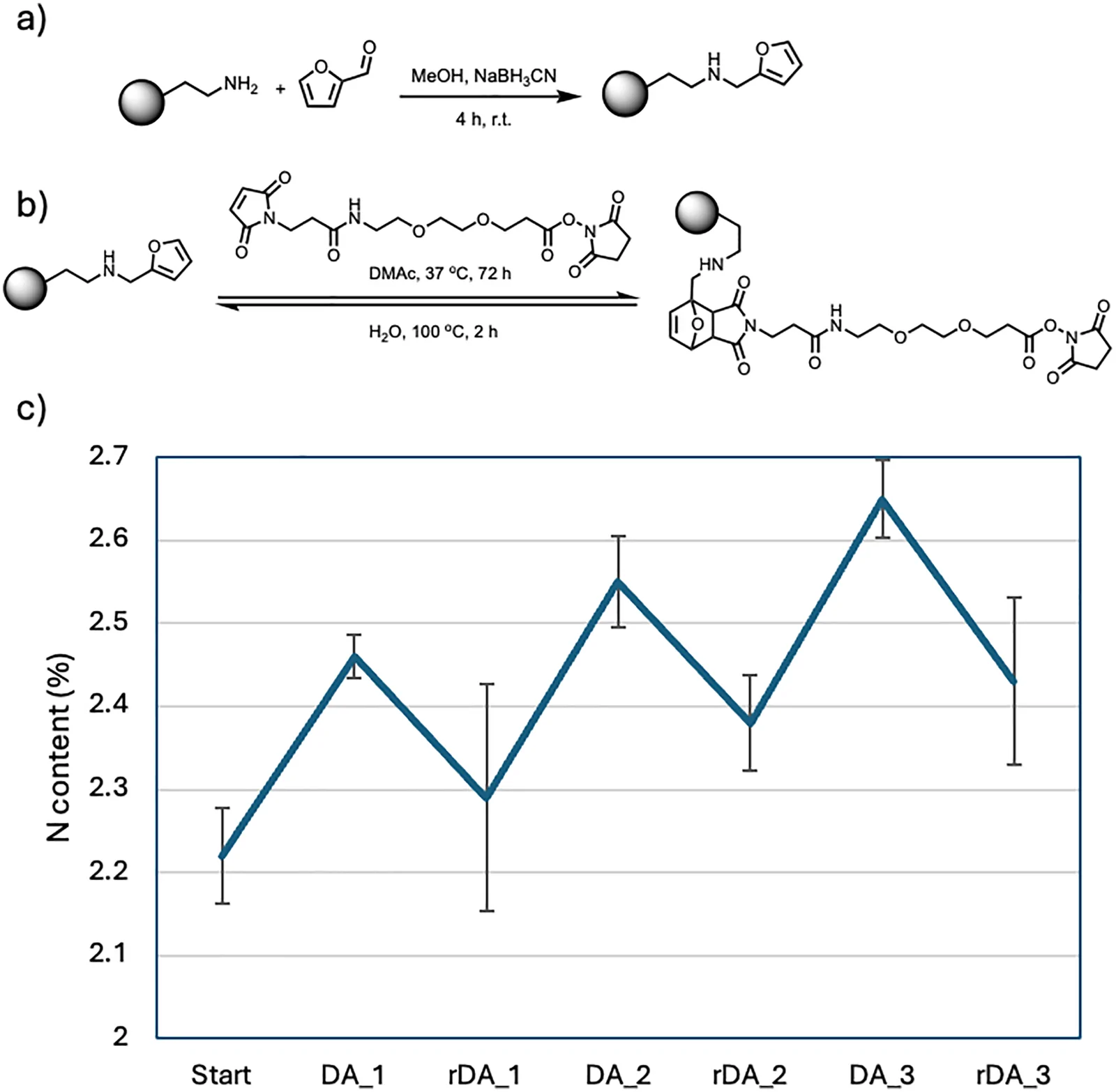
(a) Formation of furan-based methacrylate support. (b) DA, and rDA reactions between modified support and bifunctional linker. (c) Thermal elemental analysis of nitrogen content between three DA/rDA cycles.

To regenerate the support, the resin was simply boiled in water for two hours to trigger the retro-DA reaction. Elemental analysis confirmed complete linker removal under these conditions, whereas 30 min proved insufficient for full cleavage—an observation corroborated by parallel experiments on the soluble polymer system. The entire load–cleave sequence was repeated for three consecutive cycles on the same batch of resin (Fig. 1c). Measurements at 24 h showed lower nitrogen incorporation compared to 72 h, indicating that extended reaction times are needed for maximal linker installation (SI, Fig. S7). While the absolute nitrogen content showed a gradual upward trend across cycles, attributable to minor irreversible side reactions between residual maleimide and the resin (SI, Fig. S8),^21^ the incremental N% gain per DA/retro-DA cycle remained constant, confirming full reversibility of the productive pathway.

With a functional, recyclable support in hand, we evaluated its performance in enzyme immobilisation. *Candida boidinii* formate dehydrogenase (CbFDH) was selected as the initial model enzyme.^22^ Bioinformatic analysis with the CapiPy package^23^ and InSEIT protein suite^24^ indicated a high surface lysine content, favourable for reaction with the NHS esters of the activated resin. At a loading of 1 mg enzyme per gram of support, quantitative immobilisation yield (IY = 100%) was observed; however, recovered activity (RA) was below 5%. This substantial activity loss is consistent with over-attachment through multiple lysine residues, a phenomenon previously documented for CbFDH on methacrylic-based carriers.^3,25^

We therefore turned to the glycosyl hydrolase from *Halothermothrix orenii* (HorGH1) as an alternative model enzyme (SI, Fig. S9).^26,27^ To confirm the validity of the bioconjugation chemistry, HorGH1 (2 mg mL^−1^) was first reacted in solution with 5 equivalents of the MAL-PEG_2_-NHS linker for 2 h in HEPES buffer (100 mM, pH 8.0) (Fig. 2a). SDS-PAGE analysis revealed complete conversion of the native protein to a new species approximately 7 kDa higher (Fig. 2b), in good agreement with MALDI-TOF mass spectrometry (Fig. 2c, and SI, Fig. S10). Given that HorGH1 possesses 20 solvent-exposed lysine residues,^24^ this mass increase is consistent with modification of approximately 17 lysines. In addition, circular dichroism (CD) spectroscopy showed near-identical spectra for the native and PEGylated enzyme, confirming preservation of the secondary structure upon linker conjugation (SI, Fig. S11).^28^

**Fig. 2:**
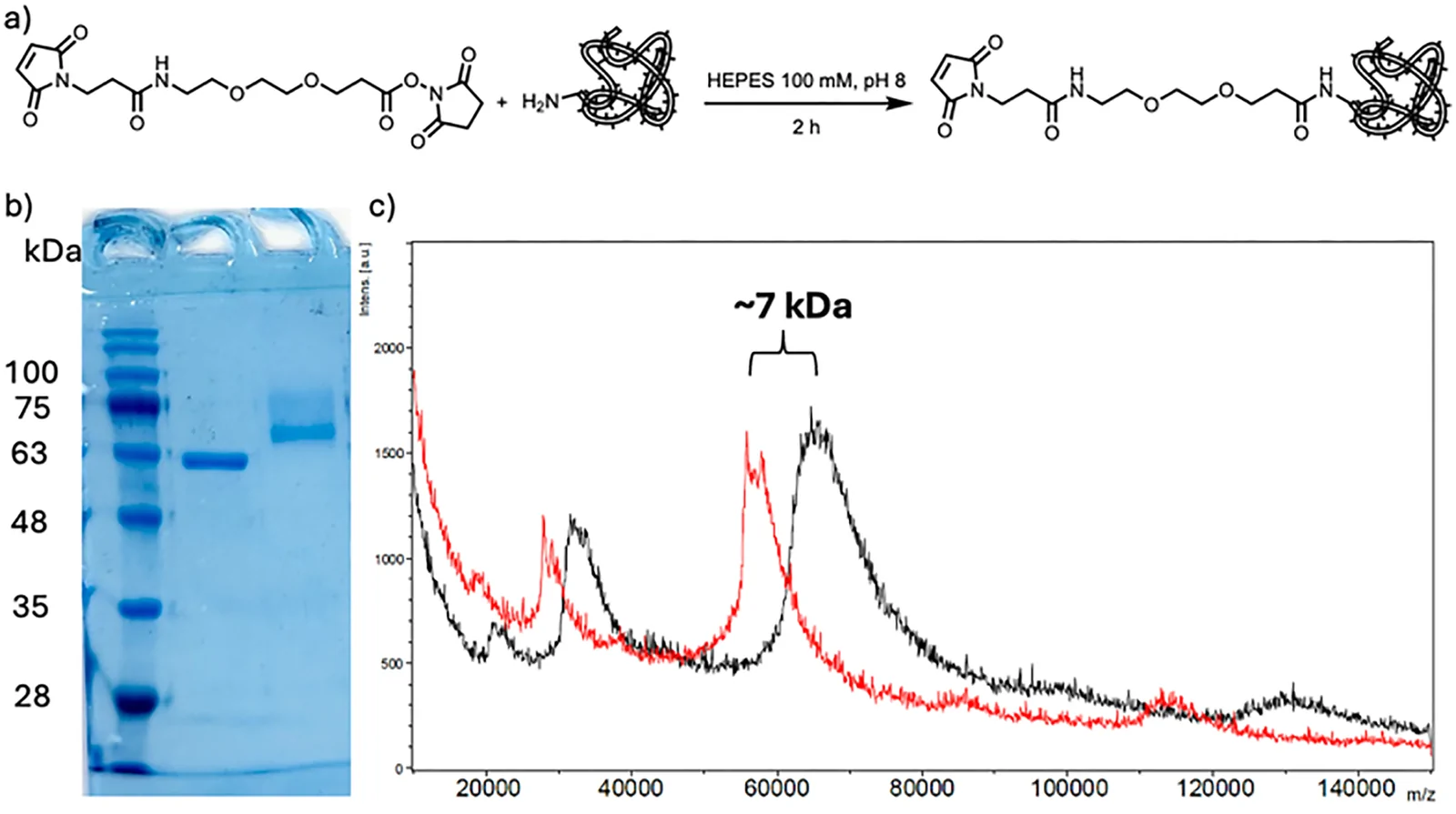
(a) Bioconjugate formation between HorGH1 and bifunctional linker. (b) Confirmation of bioconjugate *via* SDS-PAGE (lane 1 HorGH1, lane 2: HorGH1-linker adduct) and (c) MALDI-TOF of HorGH1 (red trace) and HorGH1-linker (black trace).

Immobilisation of HorGH1 on the DA-activated resin proceeded with 100% IY and a recovered activity of 60%, corresponding to an immobilised activity of 8.4 U g^−1^ (Fig. 3a). A control experiment in which the NHS groups were first blocked by reaction with glycine showed no detectable activity after washing, confirming that binding occurs exclusively through the intended NHS–lysine coupling (SI, Fig. S12). To verify that thermal cleavage of the DA adduct releases the enzyme–linker conjugate, the spent resin was boiled in water (10% SDS) and the supernatant analysed. SDS-PAGE showed a band migrating above the native HorGH1 position (Fig. 3b), consistent with the cleaved HorGH1–linker adduct, and MALDI-TOF analysis confirmed the expected mass, providing direct evidence for successful retro-DA cleavage (Fig. 3c). The indicated 3.4 kDa mass increase upon cleavage corresponds to ∼8 linker molecules being attached to each monomer of the protein (∼425 Mw per linker).

**Fig. 3:**
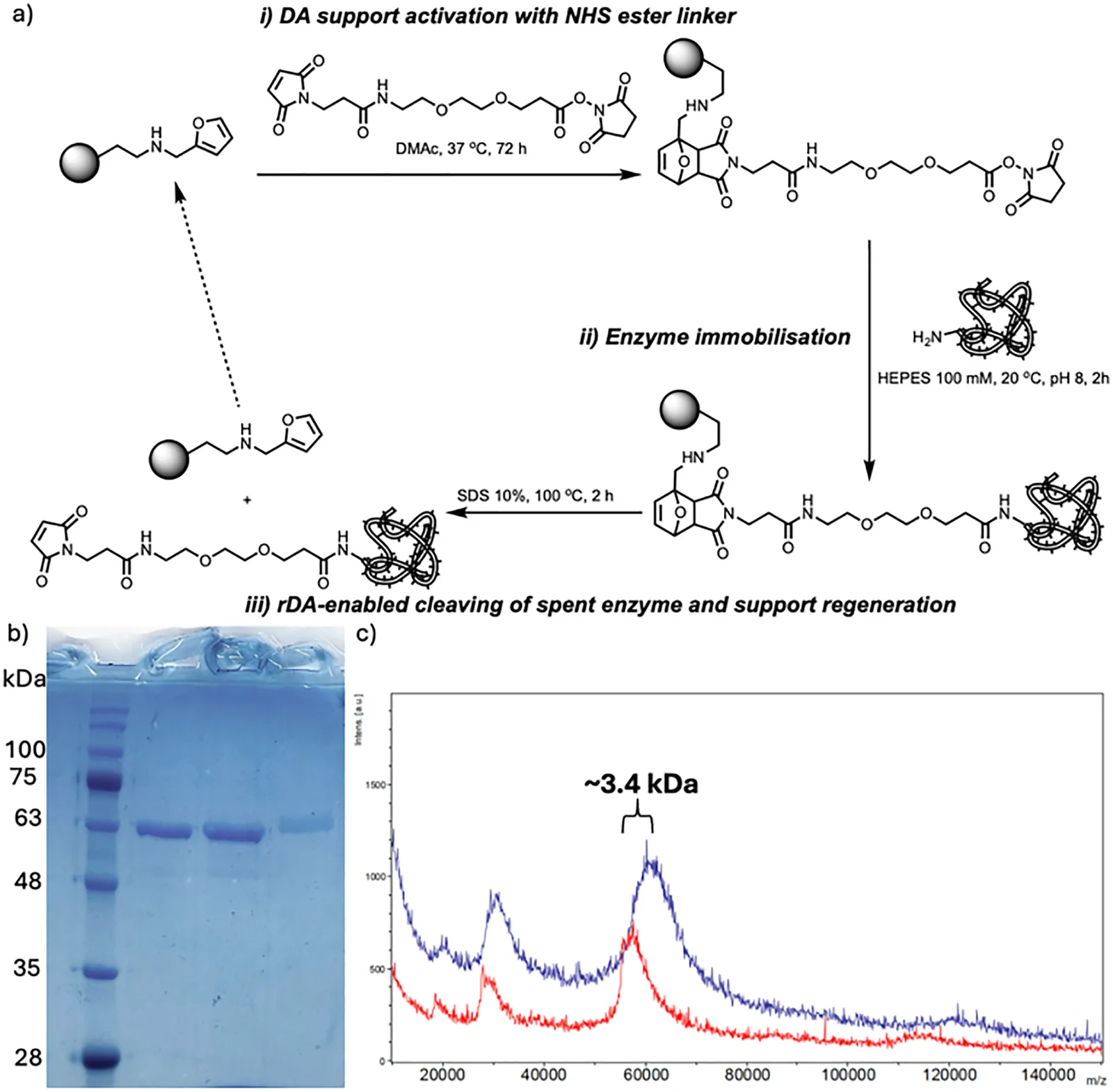
(a) Workflow showing the reversibility of the new modified support and overall reusability workflow enabled by DA/rDA chemistry. (b) SDS-PAGE gel showing: HorGH1 band (lane 1), HorGH1 after washing off blocked support (lane 2), and HorGH1 after cleaving *via* rDA (lane 3). (c) MALDI-TOF spectrum of HorGH1 (red trace) and rDA cleaved HorGH1-linker adduct (blue trace).

Finally, we assessed the operational and regeneration performance of the system over multiple cycles (Fig. 4). After immobilisation, the resin was employed in three consecutive catalytic runs using 4-nitrophenyl-β-d-glucopyranoside as a substrate (10 mM). The enzyme was then cleaved *via* rDA, a fresh linker was installed by DA reaction in DMAc (72 h), a new batch of HorGH1 was immobilised, and three catalytic cycles were carried out with the same active support. This entire load–use–cleave–reload sequence was repeated six times in total. Across all six cycles, the immobilised system retained catalytic competence with an average conversion of 27.4 ± 8.4% (Fig. 4b), and no systematic downward trend indicative of cumulative support deterioration was observed. The cycle-to-cycle variability most likely reflects gradual loss of accessible furan sites rather than enzyme deactivation, since a fresh batch of enzyme is supplied in each cycle. Even with this variability, six productive regeneration cycles represent a marked step-change over conventional covalent immobilisation, where supports are discarded after a single enzyme lifetime. The low material cost furfural for resin functionalisation and only trace quantities of the bifunctional linker (€80 g^−1^) needed per cycle, makes each regeneration economically negligible, extending carrier utilisation from one to multiple productive lifecycles.

**Fig. 4:**
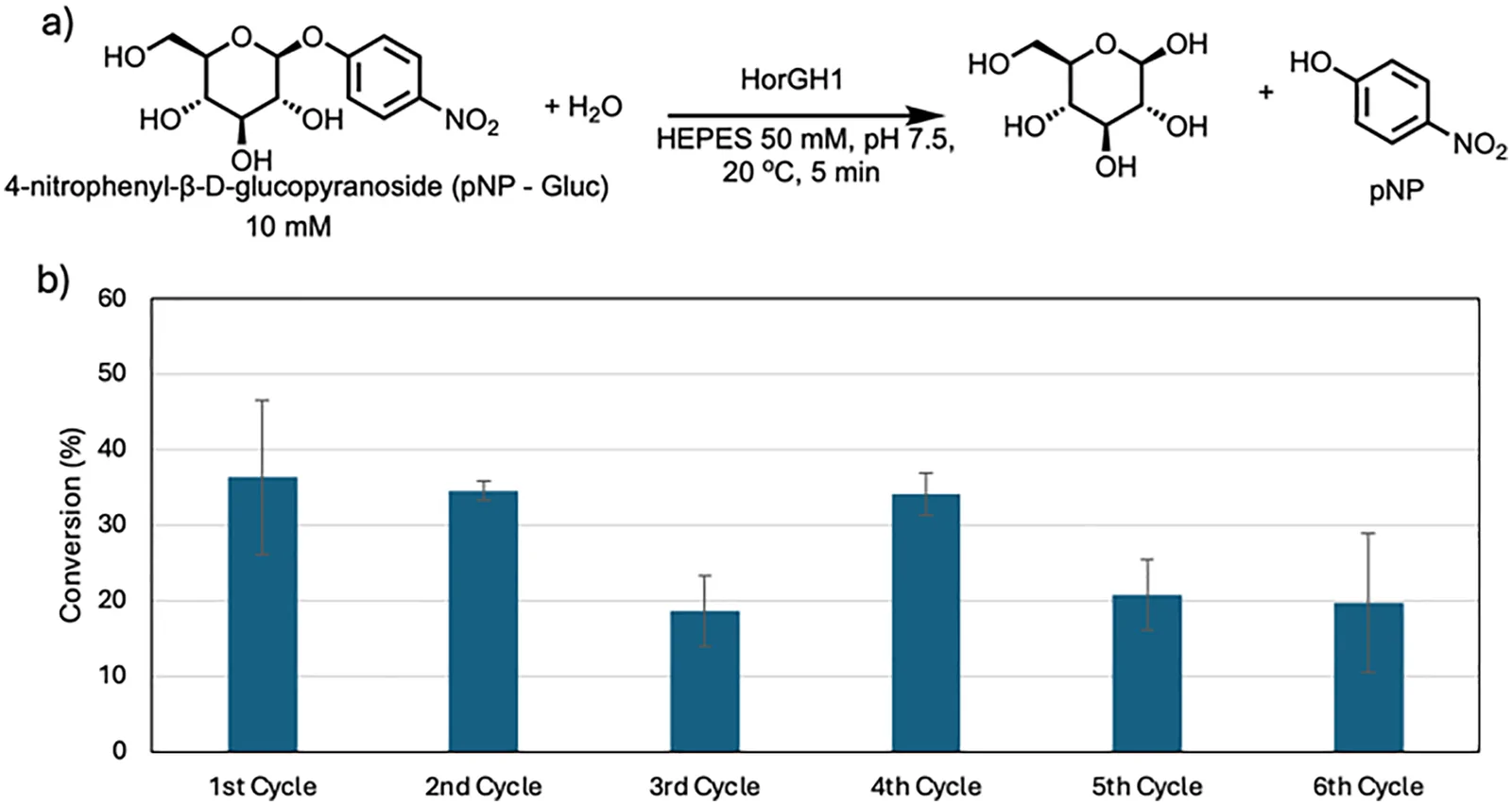
(a) Conversion of pNP-Gluc to glucose and pNP by HorGH1. (b) Use of the same immobilisation support over six different DA/rDA cycles showing reusability over the same conditions. 2 mg_protein_ g_support_^−1^ were offered at each loading cycle.

In summary, we have demonstrated that a straightforward chemical modification of a commercially available C_2_-amine methacrylate resin converts it into a reusable enzyme immobilisation support. The furan–maleimide DA/retro-DA cycle provides a thermally triggered, fully reversible linkage strategy that is compatible with standard NHS-based bioconjugation. While the cleavage treatment would not be suitable if the biocatalysts would need to maintain activity, this approach is perfect to recover and reuse the support in a fresh immobilisation cycle at the end of the enzyme lifespan.

Multiple attach–cleave cycles were achieved, offering a practical route to reduce the environmental burden of spent immobilisation carriers. Although the present work constitutes a proof of concept for a single resin type, the underlying chemistry is readily transferable: for instance, epoxy-functionalised supports could be reacted with furfurylamine to generate analogous furan-decorated carriers amenable to the same DA coupling protocol. Work is currently ongoing to extend this approach to a broader library of resins, linkers, and enzymes.

## Author contributions

E. B. and F. P. conceptualized the idea and F. P. supervised the project and obtained funding. E. B. performed the experimental work and wrote the initial draft. Both authors discussed and agreed to the final version of the manuscript.

## Conflicts of interest

There are no conflicts to declare.

## Supplementary Material

CC-062-D6CC02525K-s001

## Data Availability

The data supporting this article have been included as part of the supplementary information (SI). Supplementary information is available. See DOI: https://doi.org/10.1039/d6cc02525k.
