# Supplementary material for: Thermally reversible Diels–Alder linkers enable reusable methacrylate supports for enzyme immobilisation
Source: Chem Commun (Camb). 2026 Aug 25;62(75):18769–72. doi: 10.1039/d6cc02525k (PMC13523047; doi:10.1039/d6cc02525k)
Supplement: Supplementary file 1 [file CC-062-D6CC02525K-s001.pdf]

Supplementary Information (SI) for Chemical Communications.
This journal is © The Royal Society of Chemistry

Supporting Information

Thermally reversible Diels–Alder linkers enable reusable methacrylate supports for enzyme immobilization

Emmanouil Broumidis,[1] and Francesca Paradisi*,[1]

1. *Department of Chemistry, Biochemistry and Pharmaceutical Sciences, University of Bern, Bern 3012, Switzerland.*

Table of Contents

General considerations 3

Pictures of methacrylate microparticles 4

Synthesis of soluble polymers and characterisation 5

Model proteins tested for immobilisation on the PVBC polymer 9

Installation of furan groups on the commercial C2-amine resin 10

Ninhydrin stain 11

IR spectra 12

Forward Diels–Alder coupling of the furan-functionalised resin with MAL-PEG₂-NHS linker 13

Retro-Diels–Alder cleavage and regeneration of the support 14

Additional elemental analysis data and related calculations 15

Potential side-reactions on the modified support 16

Expression and purification of HorGH1 protein 17

Sample preparation for SDS-PAGE and MALDI-TOF analysis of rDA cleaved proteins 18

CD spectroscopy spectra 19

Immobilised HorGH1 on NHS activated, and control (blocked) supports 20

References 21

General considerations

All chemicals were commercially available except those whose synthesis is herein described. Furfural, sodium cyanoborohydride ($NaBH_3CN$), glycine, and solvents (methanol, DMAc) were purchased from Sigma-Aldrich or TCI and used as received. The MAL–PEG₂–NHS linker (CAS no: 955094-26-5) was obtained from BLDPharm. C2-amine methacrylate resin (ECR8309F) was supplied by Purolite (Ecolab). Small-scale liquid handling was performed using variable-volume (2.00–5000.00 μL) single-channel Starlab ErgoOne™ precision micropipettes. FT-IR spectra were recorded on a Jasco FT/IR-4700 spectrometer equipped with a Jasco ATRproONE accessory; spectra were collected in the range 4000–400 cm⁻^{1} at 4 cm⁻^{1} resolution. Elemental analysis (C, H, N) was carried out by the microanalytical laboratory service (ARS) at the University of Bern using a Flash 2000 Organic Elemental Analyzer (Thermo Scientific). The concentration of Gluc-pNP was determined by HPLC (Dionex UltiMate 3000, Waters X-Bridge C18 (3.5 μm, 2.1 mm × 100 mm), measuring at 210, 250, and 265 nm), using a gradient method from 5:95 to 95:5 (H₂O:MeCN, 0.1% TFA) over 10 min with a flow rate of 0.8 mL/min, and heated at 45 °C. UV-Vis measurements for enzymatic activity assays were performed on a temperature-controlled Agilent Cary-60 spectrophotometer. SDS-PAGE was run on 4～20% Bis-Tris gels under denaturing conditions and stained with Coomassie Brilliant Blue dye. MALDI-TOF mass spectra were acquired on a Bruker Autoflex instrument using 2,6-Dihydroxyacetophenone (DHAP) as the matrix. Circular dichroism (CD) spectra were recorded on a ChiraScan V100 (Applied Photophysics Ltd) spectropolarimeter using a 1 mm path-length quartz cuvette. ^{1}H NMR spectra were recorded at 300 MHz on a Bruker Avance spectrometer. Bioinformatic analysis of enzyme surface residues was performed using the CapiPy package[1] and InSEIT's protein immobilization suite.[2]

Pictures of methacrylate microparticles

a)

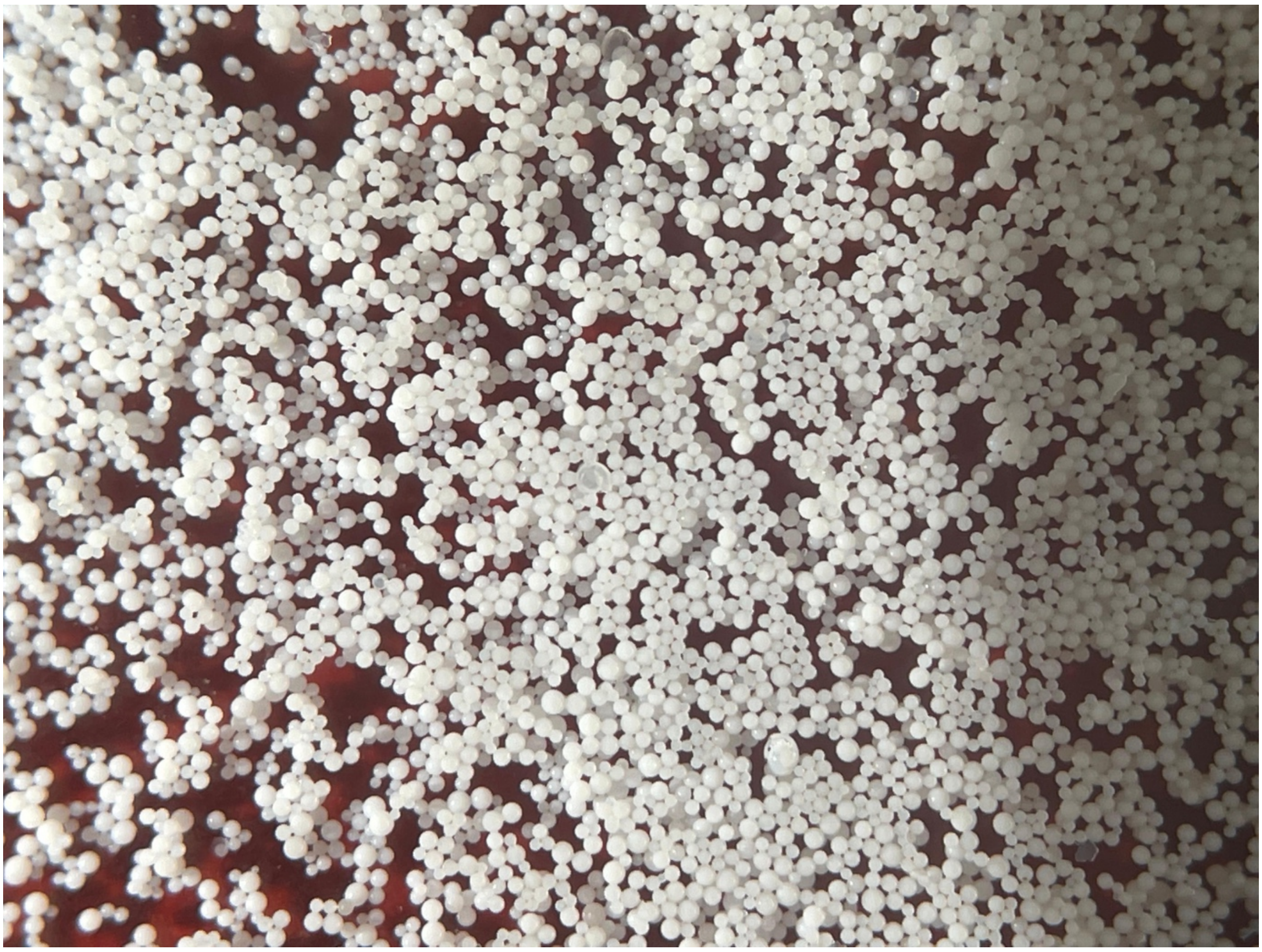

b)

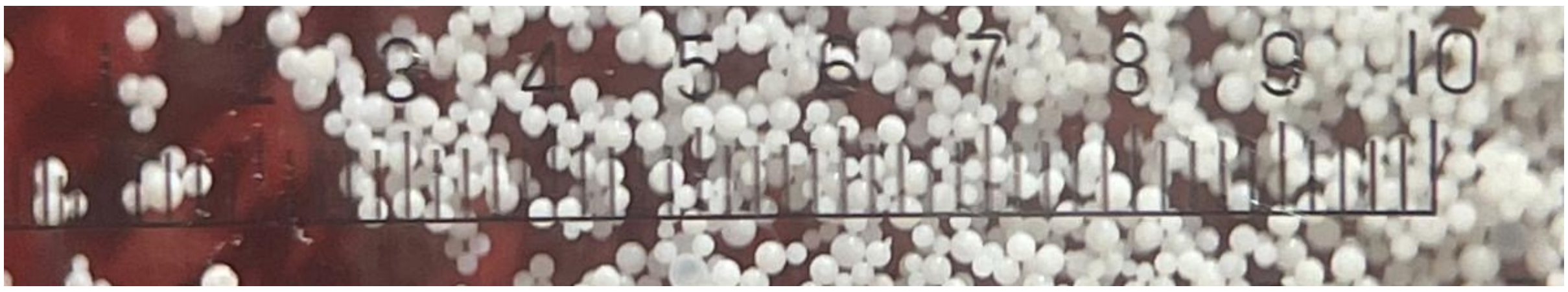

Figure S1: a) C2-amino methacrylate support used in this study under optical magnification (10x). b) Scale bar in mm. Average particle size is 100 μm.

Synthesis of soluble polymers and characterisation

General Procedure for Etherification of Poly(vinylbenzyl chloride) with Furfuryl Alcohol

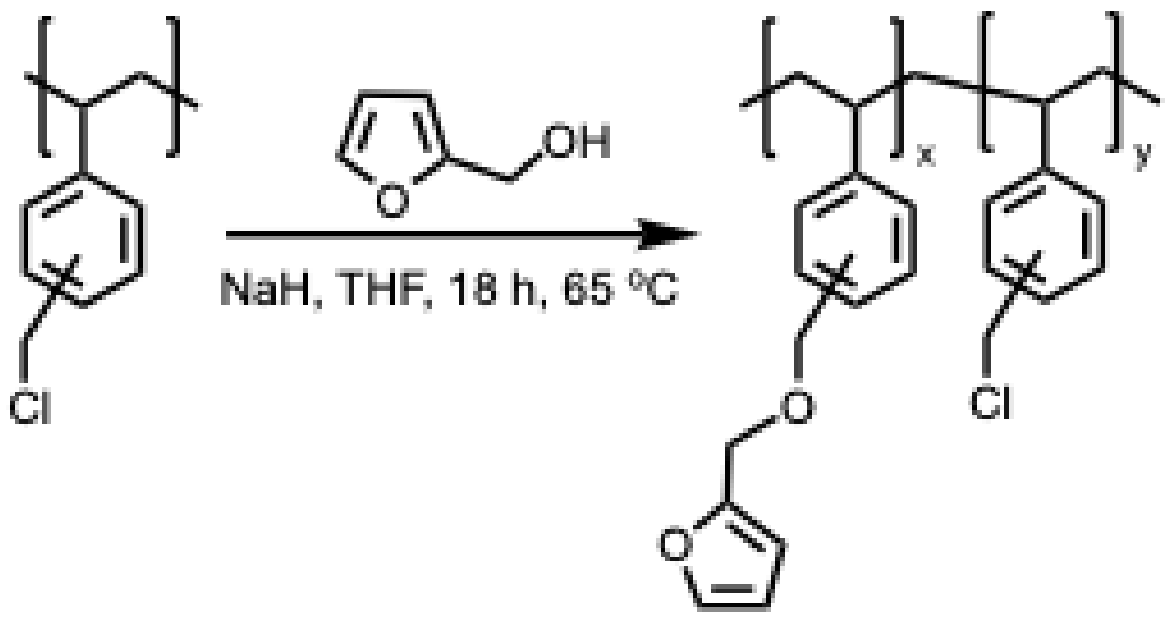

To a solution of furfuryl alcohol (0.96 g, 9.83 mmol, 1.5 equiv relative to chloromethyl units) in dry THF (20 mL) was added sodium hydride (60 wt% dispersion in mineral oil, 393 mg, 9.83 mmol, 1.5 equiv) portionwise at 0 °C under nitrogen. The mixture was allowed to warm to room temperature and stirred for 30 min until gas evolution ceased. A solution of poly(vinylbenzyl chloride) (1.0 g, 6.55 mmol chloromethyl equiv; 60/40 mixture of 3- and 4-isomers) in dry THF (10 mL) was then added dropwise, and the reaction mixture was heated to reflux for 18 h under nitrogen. After cooling to room temperature, the solution was concentrated to approximately half its volume under reduced pressure and precipitated into methanol. The product was collected by filtration, washed with methanol, and dried under vacuum at 30 °C for 24 h to afford the furan-functionalised polymer as a light-beige solid (1.1 g). ^{1}H NMR analysis confirmed partial functionalisation, with characteristic furan ring protons (δ 7.39–7.44 ppm) clearly resolved alongside residual chloromethyl signals (–CH₂Cl, δ ~4.36-4.49 ppm), indicating incomplete conversion of the benzylic chloride groups. The degree of substitution was estimated at 21% by integration of the furfuryl methylene relative to the residual –CH₂Cl resonance.

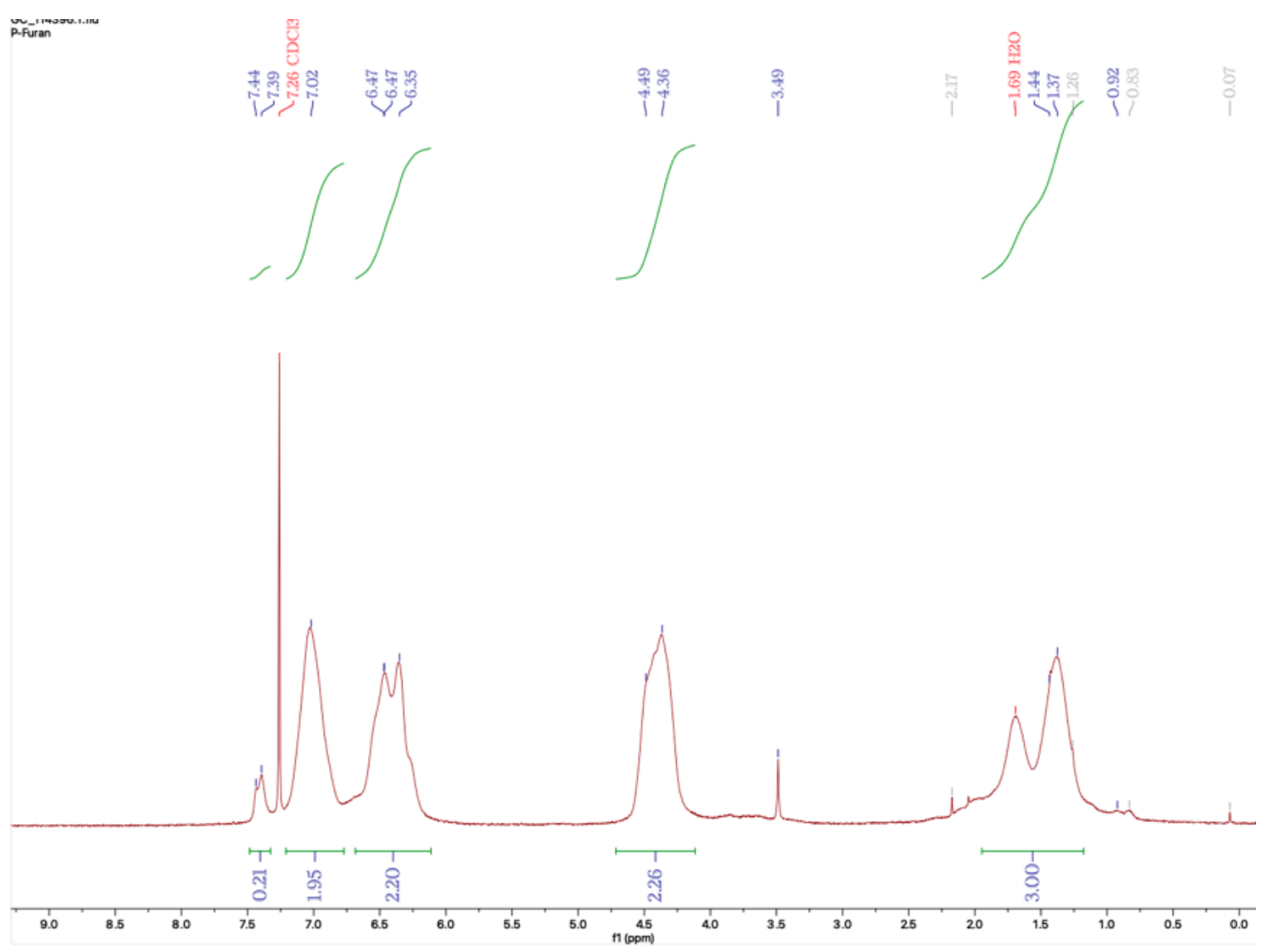

Figure S2: 1H-NMR spectrum of substituted PVBC polymer.

General Procedure for Diels–Alder Conjugation of Furan-Functionalised Poly(vinylbenzyl chloride) with Maleimide-PEG₂-Succinimidyl Ester

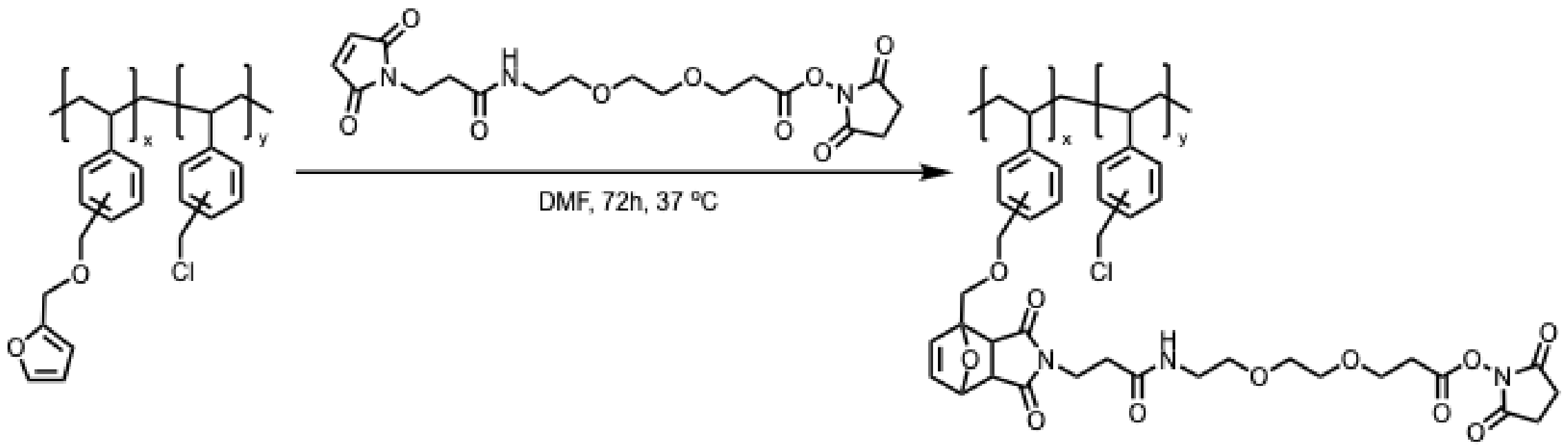

A solution of maleimide-PEG₂-succinimidyl ester (1.5 equiv relative to furan units, as determined by ^{1}H NMR of the furan-functionalised polymer) in dry DMF (2.5 mL) was added to a solution of furan-functionalised poly(vinylbenzyl chloride) (500 mg, 1.0 furan equiv) in dry DMF (2.5 mL). The mixture was purged with nitrogen for 15 min, sealed, and stirred at 37 °C for 72 h in the dark. After cooling to room temperature, the reaction mixture was precipitated into methanol (50 mL). The solid was collected by filtration, washed thoroughly with methanol to remove unreacted linker, and dried under vacuum at room temperature for 24 h to afford the DA-functionalised polymer as an amber-coloured solid (530 mg).

^{1}H NMR analysis confirmed successful cycloaddition through the appearance of the characteristic oxanorbornene bridgehead protons (δ ~5.2–5.3 ppm) and an amide resonance at δ ~7.92 ppm, with concomitant attenuation of the furan signals (7.55 ppm), consistent with [4+2] cycloaddition across the pendant furan groups. Residual furan resonances indicated incomplete conversion, potentially due to crowding effects.

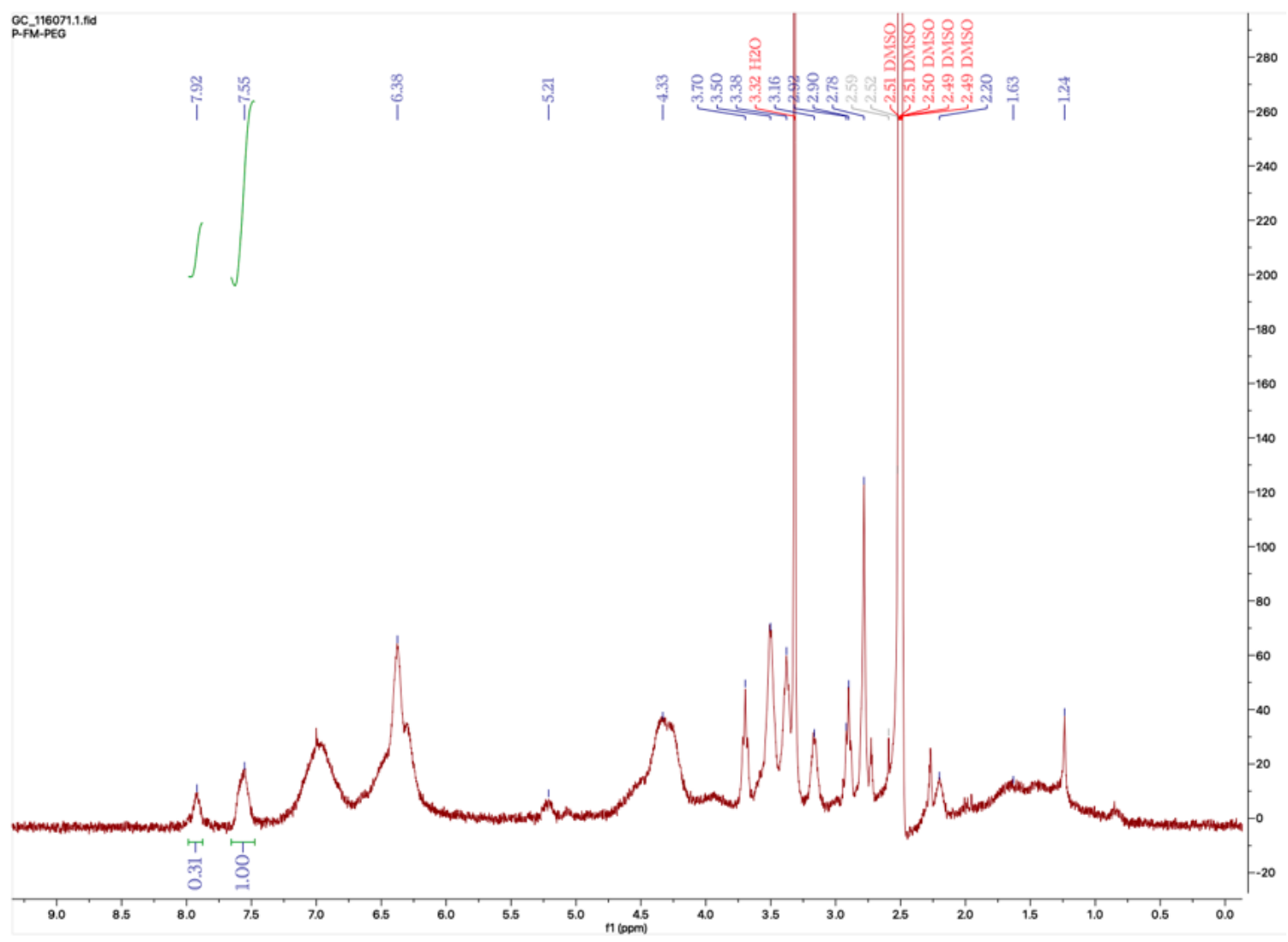

Figure S3: 1H-NMR spectrum of DA polymer product.

Thermal Reversibility of the Diels–Alder Adduct Monitored by DOSY NMR

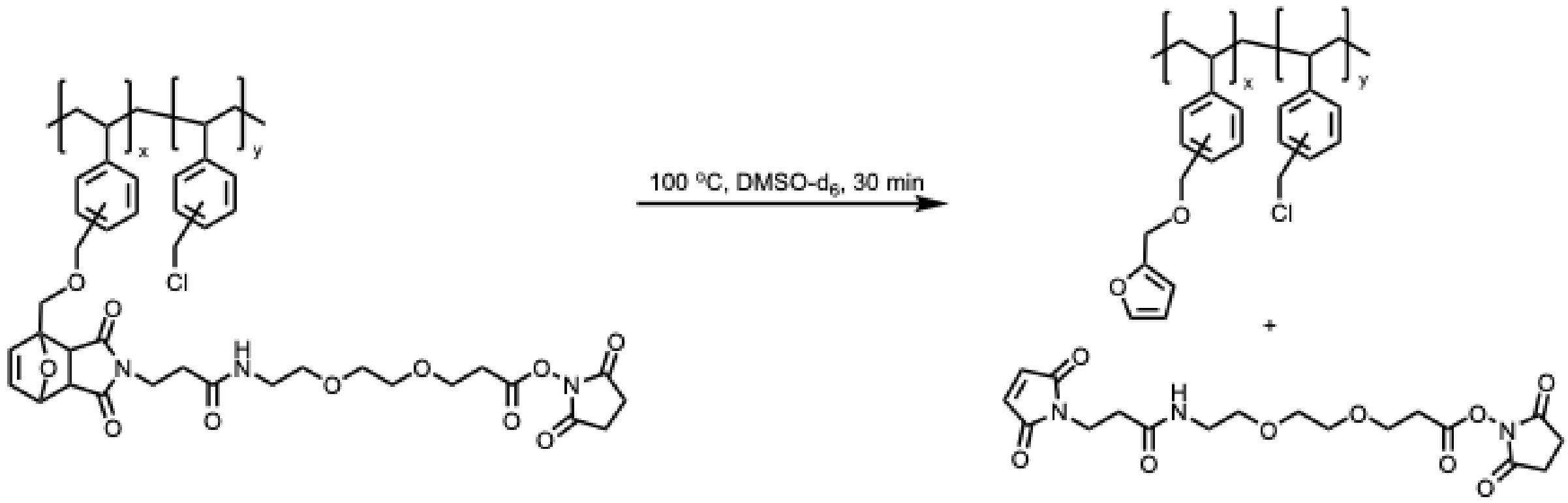

The thermal reversibility of the furan–maleimide cycloadduct was assessed by diffusion-ordered spectroscopy (DOSY). A sample of the DA-functionalised polymer was dissolved in DMSO-d₆ and an initial DOSY spectrum was recorded, showing a single diffusion band consistent with the polymer-bound PEG linker (D=4.5 x 10^{-7} cm^2/sec). The NMR tube was then heated at 100 °C for 30 min to promote the retro-Diels–Alder reaction and allowed to cool briefly before a second DOSY spectrum was acquired. The post-heating spectrum revealed the appearance of a distinct, faster-diffusing band (D = 9.5 x 10^{-5} cm^2/sec) corresponding to the maleimide-PEG₂-succinimidyl ester, alongside the slower-diffusing polymer signal (D = 1.1 x 10^{-7} cm^2/sec). The diffusion coefficient of the released species matched that of an authentic sample of the PEG linker, confirming that the oxanorbornene adduct undergoes clean thermal cleavage under these conditions. These results demonstrate that the furan–maleimide linkage is fully thermally reversible, establishing the basis for on-demand linker release and support regeneration.

a) b)

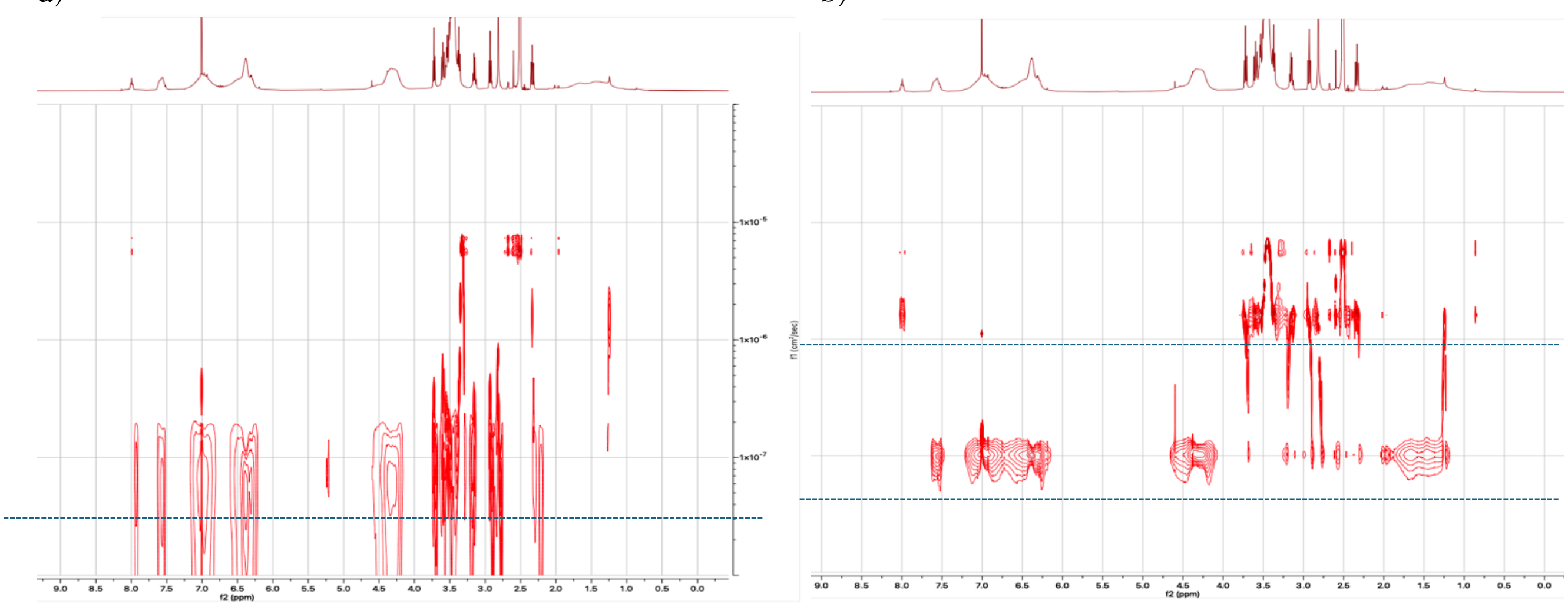

Figure S4: DOSY NMR comparison between a) DA adduct, and b) rDA adduct of polymeric sample after heating at 100 °C for 30 min.

Model proteins tested for immobilisation on the PVBC polymer

Four structurally diverse proteins were screened as candidates on the soluble PVBC–DA system. (i) Formate dehydrogenase from the yeast Candida boidinii (CbFDH, EC 1.17.1.9), which catalyses the NAD⁺-dependent oxidation of formate to CO₂ and is widely employed for cofactor regeneration in biocatalytic cascades.[3] (ii) A thermostable β-glucosidase from the halothermophile Halothermothrix orenii (HorGH1, GH1 family, EC 3.2.1.21), which hydrolyses β-1,4-glycosidic bonds in glucosides and oligosaccharides.[4] (iii) miniSOG (mSOG), a 106-residue flavin-binding fluorescent protein engineered from the LOV2 domain of Arabidopsis thaliana phototropin 2, which fluoresces and photogenerates singlet oxygen under blue-light excitation.[5] (iv) Bovine serum albumin (BSA), the 66 kDa plasma transport protein from Bos taurus, routinely used as a generic model substrate in bioconjugation and protein-binding studies.[6]

Installation of furan groups on the commercial C2-amine resin

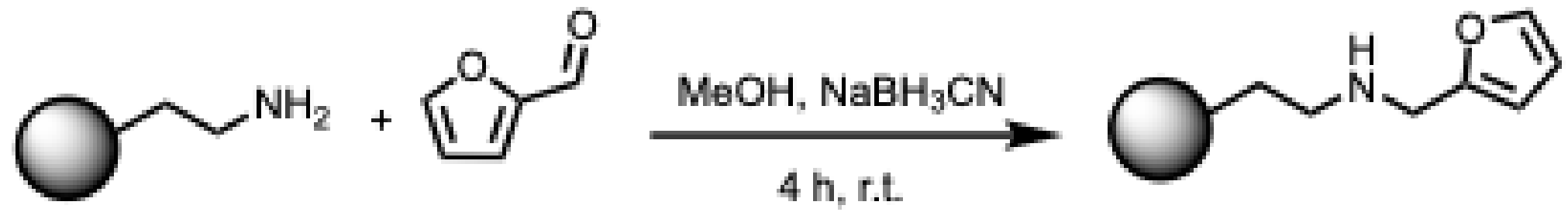

C2-amine methacrylate resin (1.00 g, 1.7 mmol g^{-1} primary amine loading, 1.7 mmol NH_2) was placed in a 20 mL polypropylene syringe fitted with a frit and suspended in methanol (5 mL). Furfural (0.70 mL, 817 mg, 8.5 mmol, 5 eq. with respect to surface amines) was added, followed by sodium cyanoborohydride (1.07 g, 17 mmol, 10 eq.) and glacial acetic acid (3 drops, catalytic). The syringe was sealed, placed horizontally on an orbital shaker, and agitated at room temperature for 4 h; the plunger was briefly withdrawn every 30 min to vent the evolved H_2 and prevent pressure build-up. After 4 h, the reaction mixture was drained through the frit and the resin washed with methanol (20 mL). The furan-functionalised resin was stored in the syringe at 4 °C until further use.

Ninhydrin stain

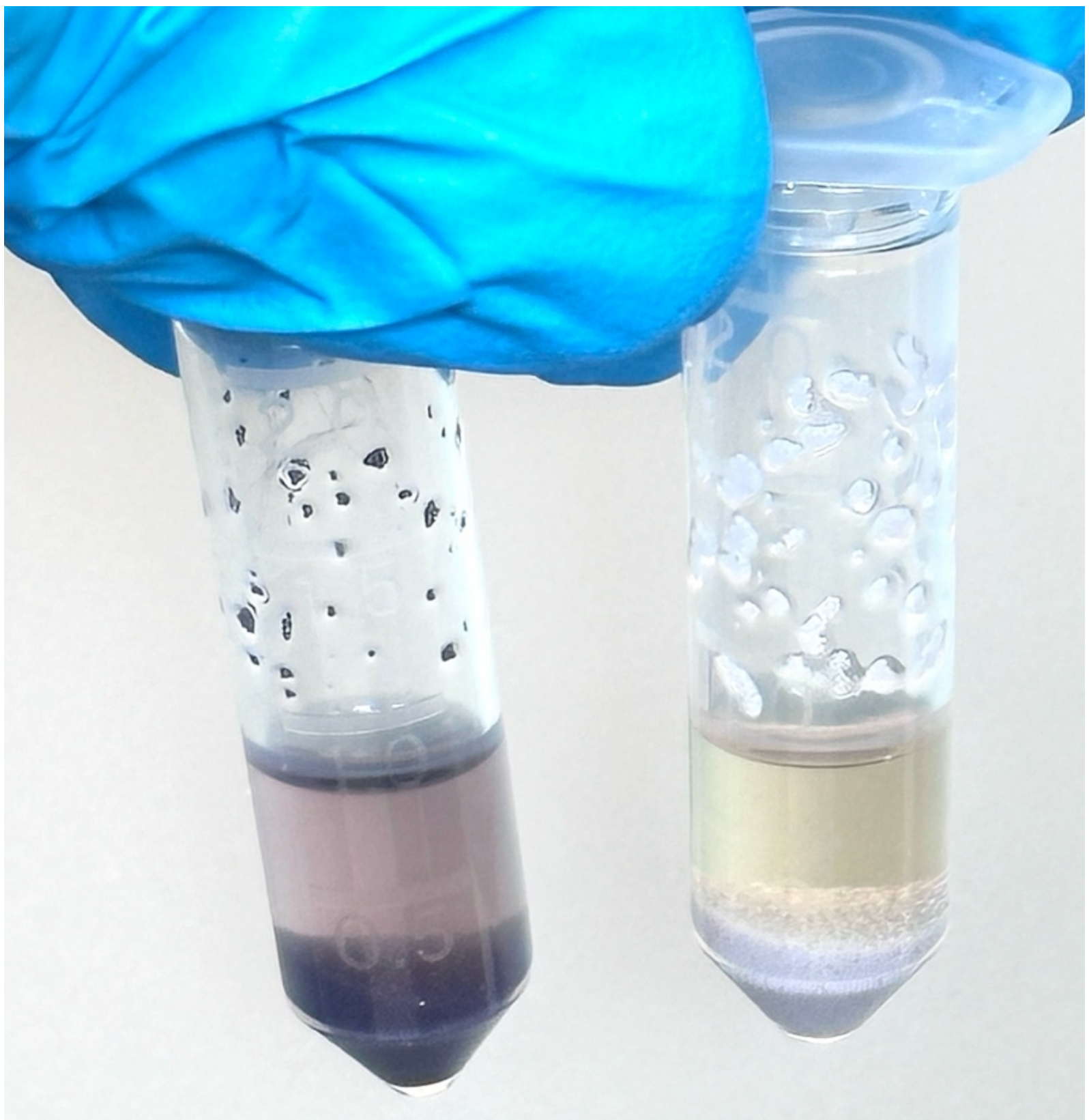

Figure S5: Ninhydrin stain test[8] of C2-NH_2 (commercial resin, left) and modified C2-NH-Furan resin (right). It is evident that only the free primary amine of the commercial sample turns a deep purple colour, while the modified resin (right), containing secondary amines only, gives a very minor response.

IR spectra

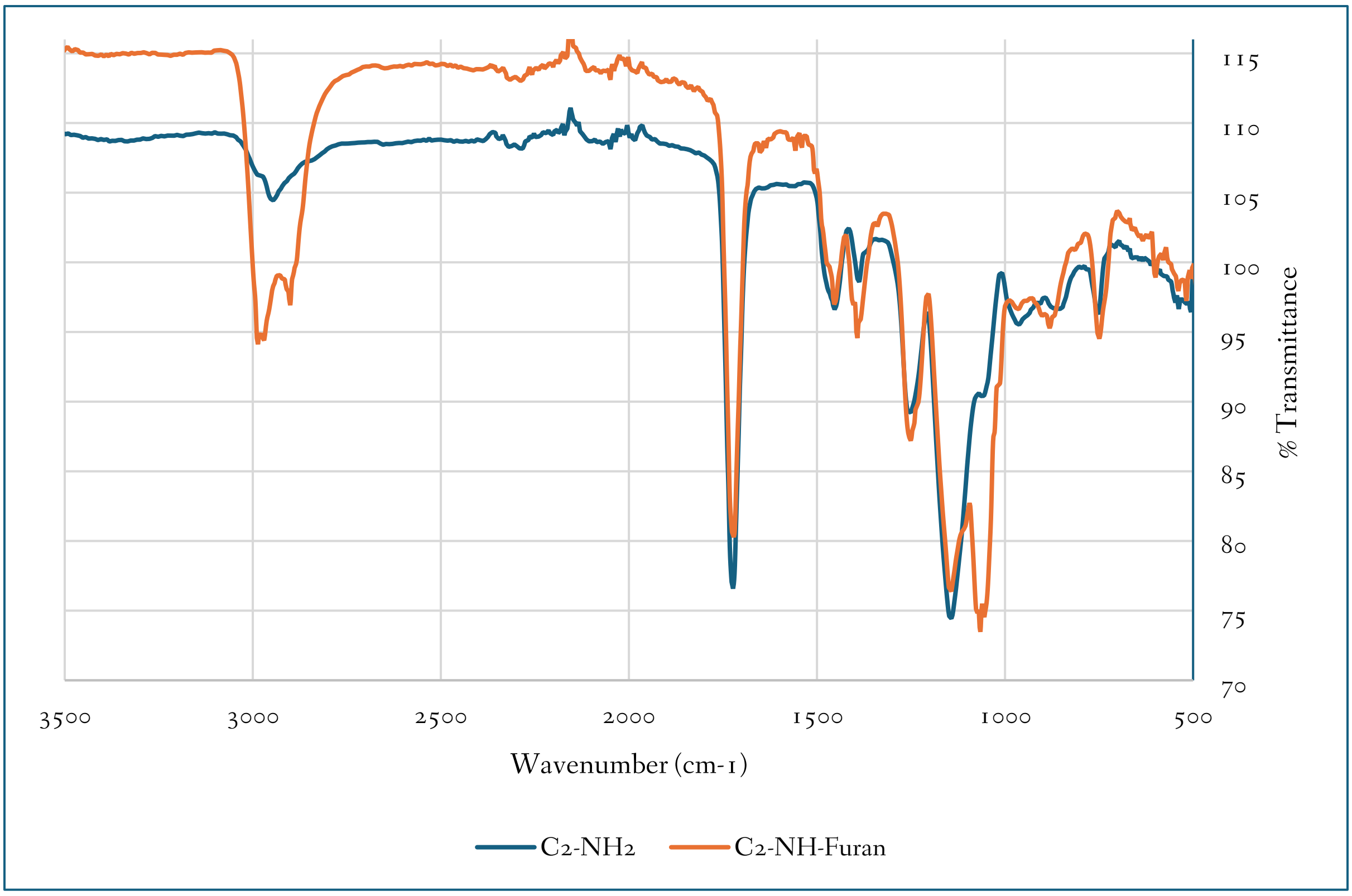

Figure S6: FT-IR spectra comparison between C2-NH2 (commercial) methacrylate resin and the modified C2-NH-Furan resin.

Forward Diels–Alder coupling of the furan-functionalised resin with MAL-PEG₂-NHS linker

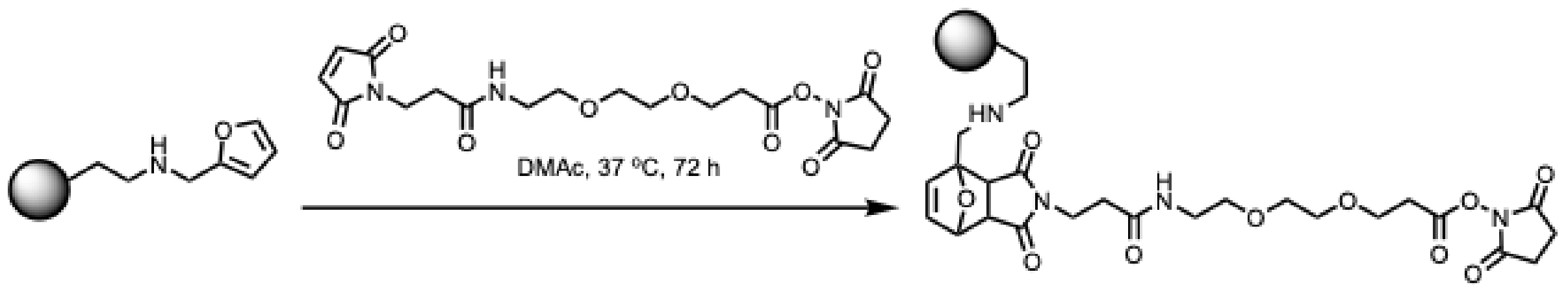

Furan-functionalised C2 methacrylate resin (100 mg) was transferred into a 5 mL polypropylene syringe fitted with a frit and washed with anhydrous DMAc (5 mL) to remove residual methanol from the preceding step. MAL-PEG₂-NHS linker (10 mg, 23.5 μmol, MW 425.4 g mol⁻^{1}; corresponding to ∼4 eq. relative to the accessible furan sites, as inferred from the saturation loading of ∼60 μmol g⁻^{1} determined by elemental analysis) was then added as a solid directly into the syringe, followed by anhydrous DMAc (1 mL). The syringe was sealed with the plunger on one end and a Luer cap on the other, placed horizontally in an orbital shaker–incubator (as used for bacterial cultures), and agitated at 37 °C for 72 h in the dark. After the reaction period, the supernatant was expelled through the frit by depressing the plunger. The resin was then washed with water (10 mL) to remove residual DMAc and unreacted linker (which is freely water-soluble), affording the NHS-activated, DA-coupled resin ready for enzyme immobilisation without further treatment. The relatively large excess of linker ensures that installation is not stoichiometry-limited; higher loadings (>72 h or additional linker equivalents) did not translate into further increases in nitrogen content by elemental analysis, indicating that the reaction is steric- rather than mass-limited under these conditions.

Retro-Diels–Alder cleavage and regeneration of the support

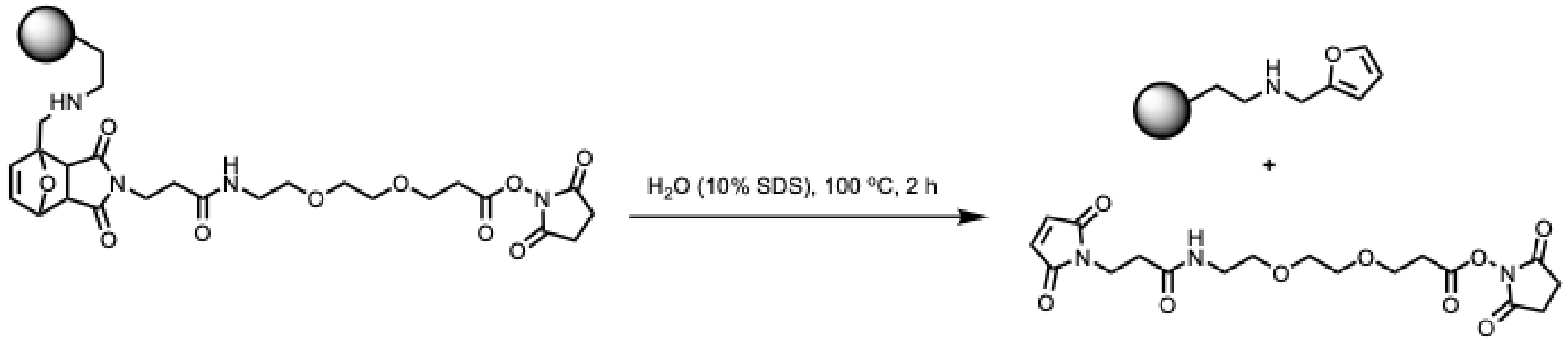

The DA-functionalised resin (100 mg, either bearing the NHS-activated linker or an immobilised enzyme–linker conjugate) was placed in a 5 mL polypropylene syringe equipped with a frit. A Luer cap was fitted to the syringe outlet and the plunger was kept removed from the top to vent water vapour during heating. Water (3 mL) was added directly into the open barrel. Where the support carried an immobilised protein, 10% (w/v) aqueous SDS was used in place of plain water to aid solubilisation and removal of the unfolded protein–linker conjugate released upon cleavage. The syringe was then positioned upright in a sand bath pre-heated to 100 °C and incubated for 30 min. Subsequently, the syringe was removed from the sand bath, the plunger was re-inserted, the outlet cap removed, and the supernatant expelled through the frit (caution: hot liquid and vapour). A fresh 3 mL aliquot of water (or 10% SDS, as appropriate) was then added and the heating cycle repeated. In total, four consecutive 30 min cycles were performed (4 × 30 min = 2 h cumulative heating) to ensure complete retro-Diels–Alder cleavage of the linker from the support. Where SDS had been used, the resin was subsequently rinsed with deionised water (5 × 5 mL) to remove all traces of detergent. In all cases, a final wash with anhydrous DMAc (5 mL) was performed to condition the regenerated resin for a subsequent forward DA reaction. The recovered furan-decorated support could be re-armed directly following the forward DA protocol above, without any intermediate treatment.

Additional elemental analysis data and related calculations

For the cycling experiments shown in the main manuscript, Figure 1c:

After the first DA coupling reaction we observe:

extra N mass = 0.0026 g N per g resin

3 N atoms per linker molecule

Moles of PEG/g_{resin} = 0.0026/(3 x 14.0067)

Moles of PEG/g_{resin} = 61.9 μmol (50-100 typical)

Furthermore, for each DA/rDA cycle we see that the amount of N gained/lost is constant:

ΔNwt%$_1$ = 0.24 %

ΔNwt%$_2$ = 0.26 %

ΔNwt%$_3$ = 0.27 %

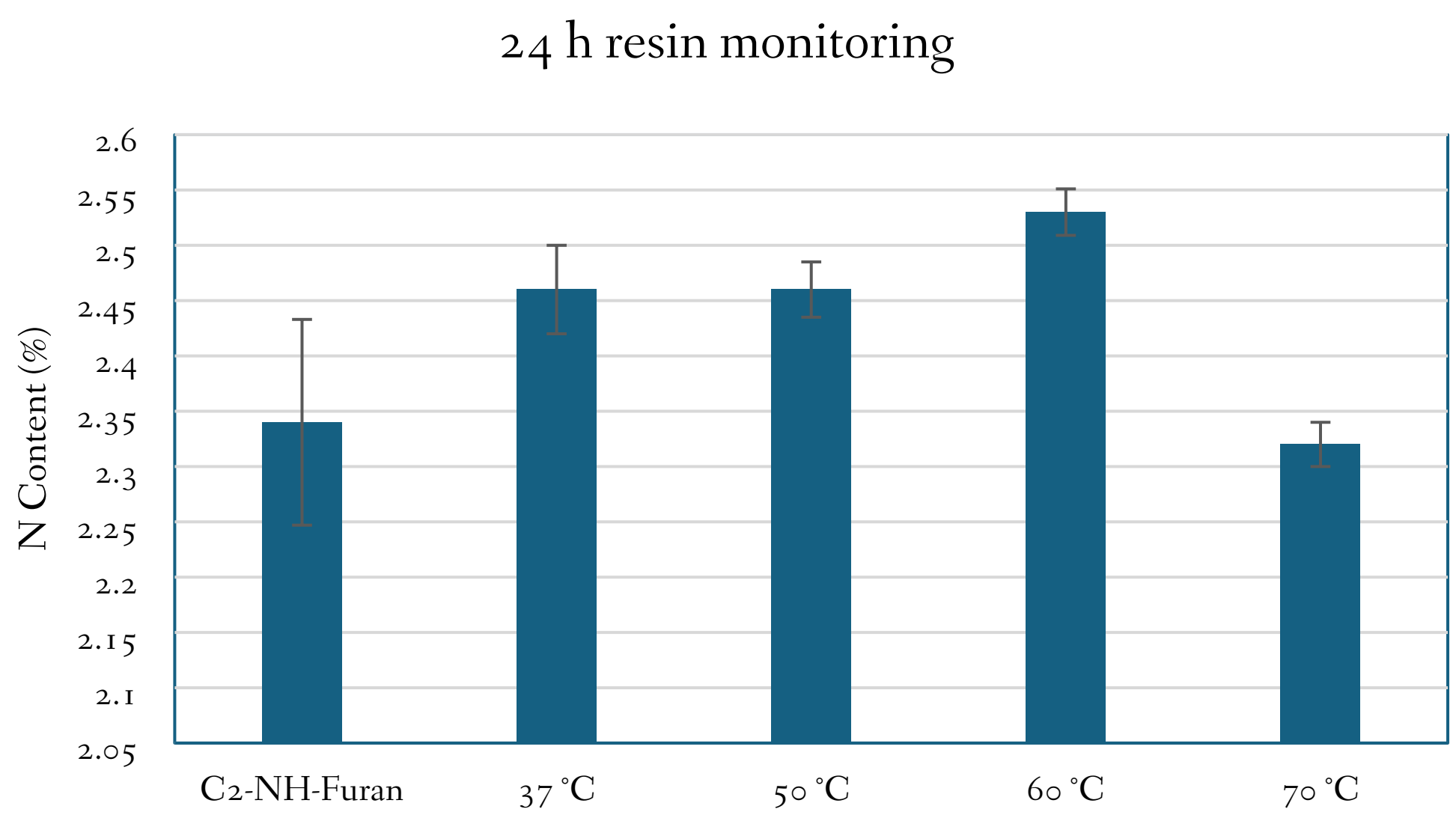

Figure S7: Elemental analysis of C2-NH-Furan resin at different temperatures, after 24 h. We observe that between 37 and 50 °C, the N wt gain is 0.12%, about half of that observed at 72 hours. Moreover, at 60 °C, the solution turned colour, from clear to red, indicating potential decomposition of the linker. At even higher temperatures (70 °C), the rDA reaction is initiated, reducing the N content gained.

Potential side-reactions on the modified support

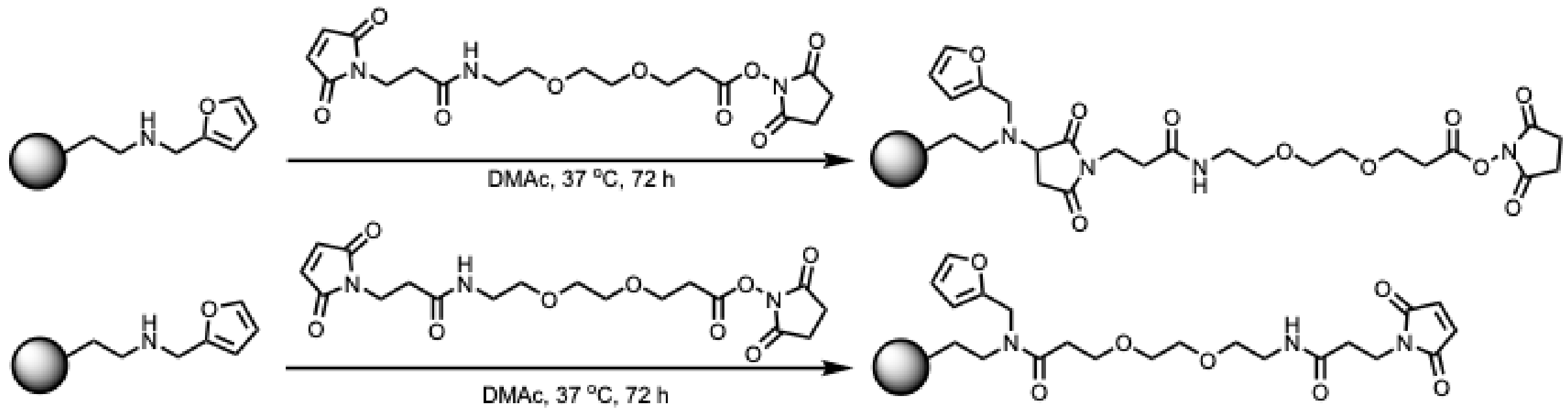

Figure S8: Two potential non-reversible side-reactions with the furan-modified support. (Top) Aza-Michael addition of the support's secondary amine to the maleimide moiety, which would trap the linker through an irreversible C–N bond and leave the NHS ester pendant.[9] (Bottom) Direct reaction of the secondary amine by the NHS ester, forming a tertiary amide and leaving a free maleimide on the support. Both pathways are expected to be minor under our conditions (DMAc, dry, 37 °C, 72 h). Although the support bears a reactive nitrogen, it is a sterically encumbered secondary amine whose nucleophilicity and accessibility toward bulky electrophiles such as NHS esters are substantially reduced relative to primary amines.[10]

Expression and purification of HorGH1 protein

HorGH1 was expressed in E. coli BL21(DE3) and purified by IMAC following the protocol reported by Pillet et al.[7] Protein purity was confirmed by SDS-PAGE and concentration was determined spectrophotometrically at 280 nm (ε = 106,230 $L·mol^{-1}·cm^{-1}$). Loading buffer was: 50 mM HEPES, 150 mM NaCl, 10 mM imidazole, pH 7.5, while elution buffer was: 50 mM HEPES, 150 mM NaCl, 300 mM imidazole, pH 7.5.

a)

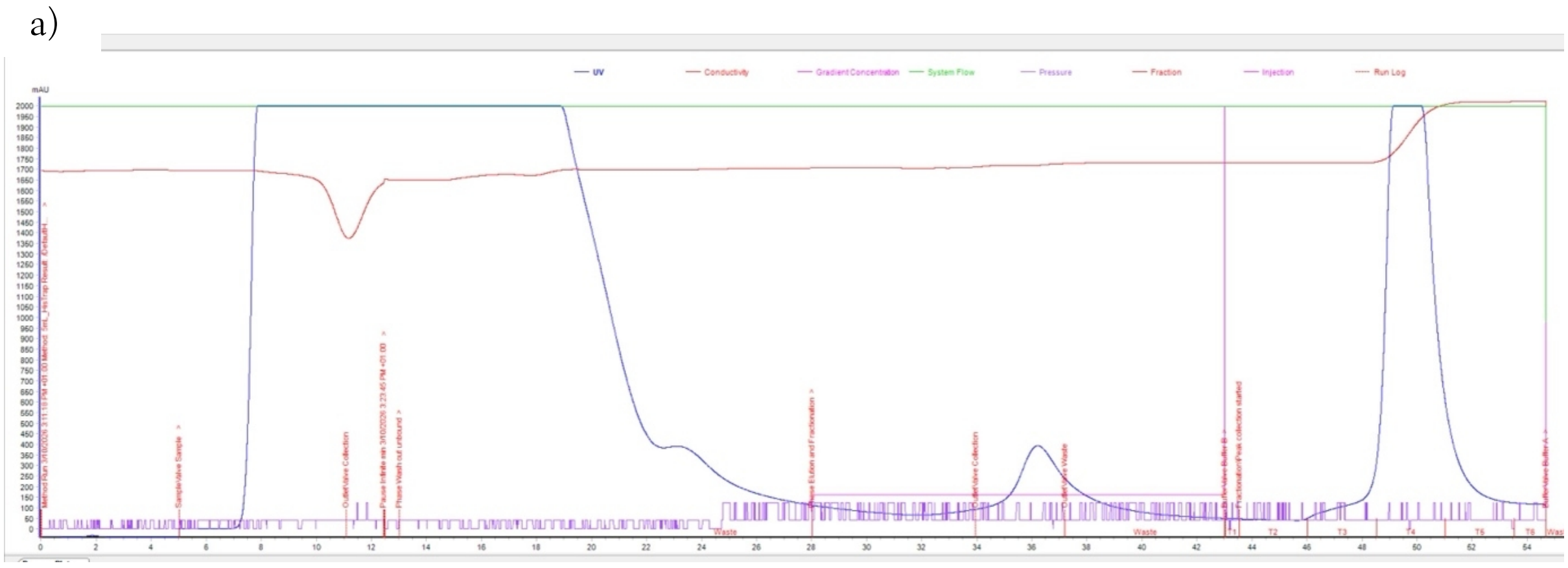

b)

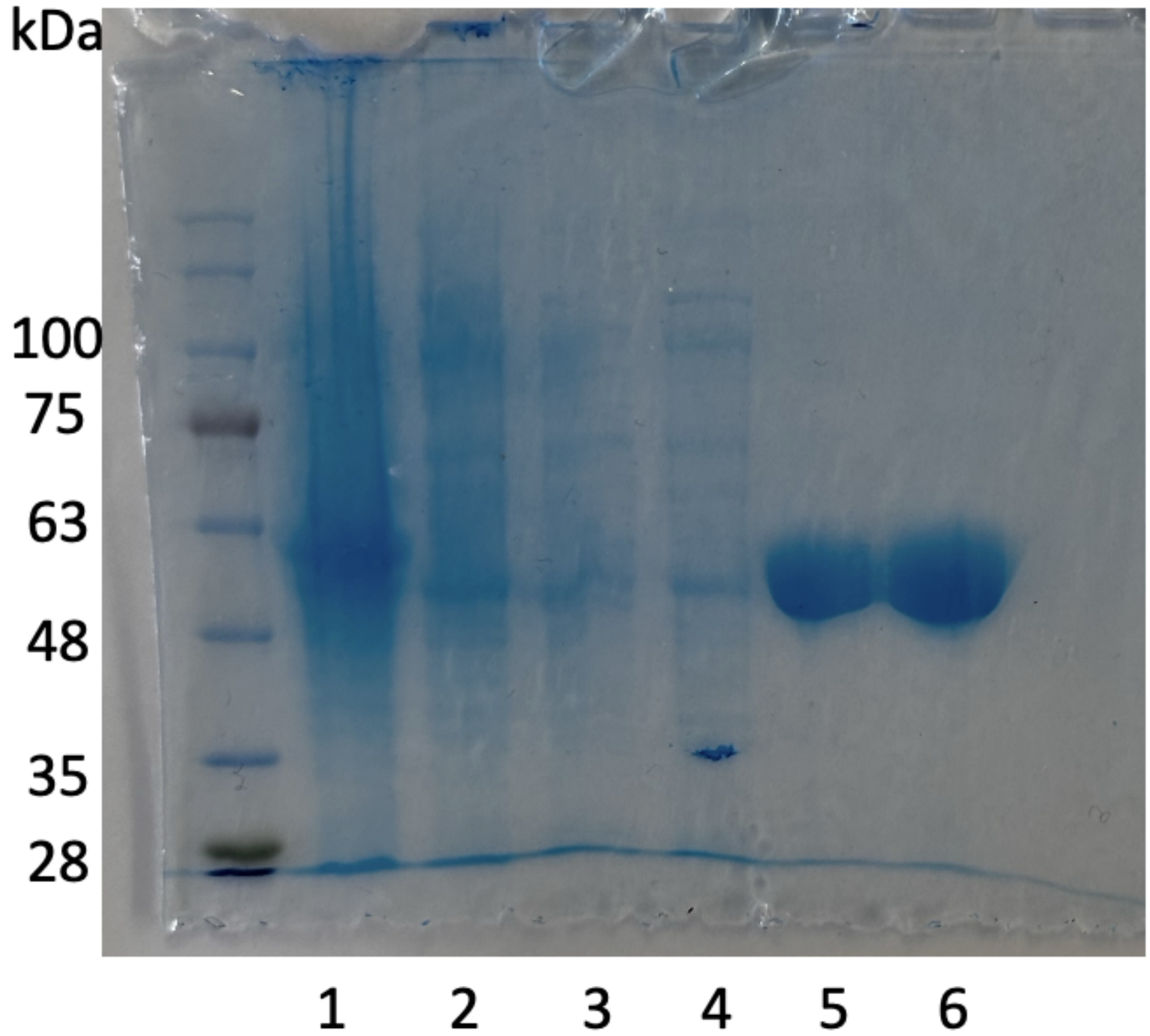

Figure S9: a) AKTA chromatogram of the purification process. Peak at ~50 min corresponds to elution with 100% elution buffer. b) SDS-PAGE of the purification process: lane 1: crude lysate. Lane 2: wash unbound (fraction 1). Lane 3: wash unbound (fraction 2). Lane 4: wash with 90% loading buffer and 10% elution buffer. Lanes 5, 6: Washing with Elution buffer. Protein yield: 42.5 $mg/L_{culture}$. Specific activity: 7.3 U/mg.

Sample preparation for SDS-PAGE and MALDI-TOF analysis of rDA cleaved proteins

The immobilised enzyme on the DA-activated support (100 mg) was placed in a 5 mL syringe equipped with a frit. To release the immobilised protein, the resin was boiled directly in SDS-PAGE loading dye (100 μL) and the resulting solution was filtered through the frit. The filtrate was used directly for SDS-PAGE analysis. For MALDI-TOF characterisation, the filtrate was diluted with ethanol (900 μL) to precipitate the bioconjugated protein. The suspension was centrifuged to pellet the protein, and the pellet was washed with ethanol (500 μL, 2×). The washed pellet was dissolved in formic acid (85% in water, 15 μL) and analysed by MALDI-TOF mass spectrometry. The matrix that was selected for analysis was 2′,6′-Dihydroxyacetophenone (DHAP).

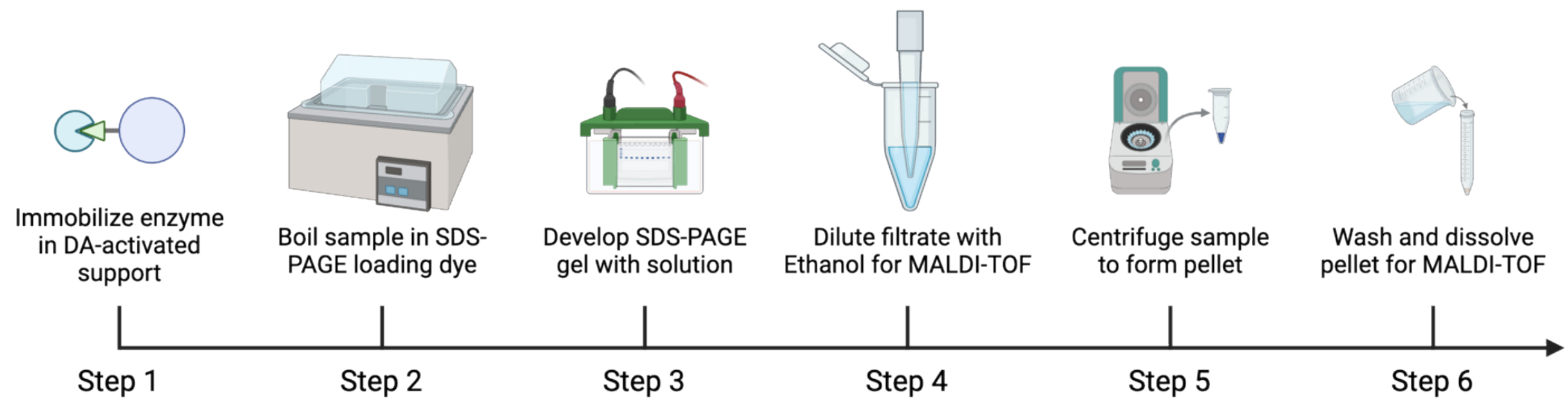

Figure S10: Workflow chart depicting the process described in the text above. Step 3 is optional (only relevant if obtaining an SDS-PAGE sample of the cleaved protein is important).

CD spectroscopy spectra

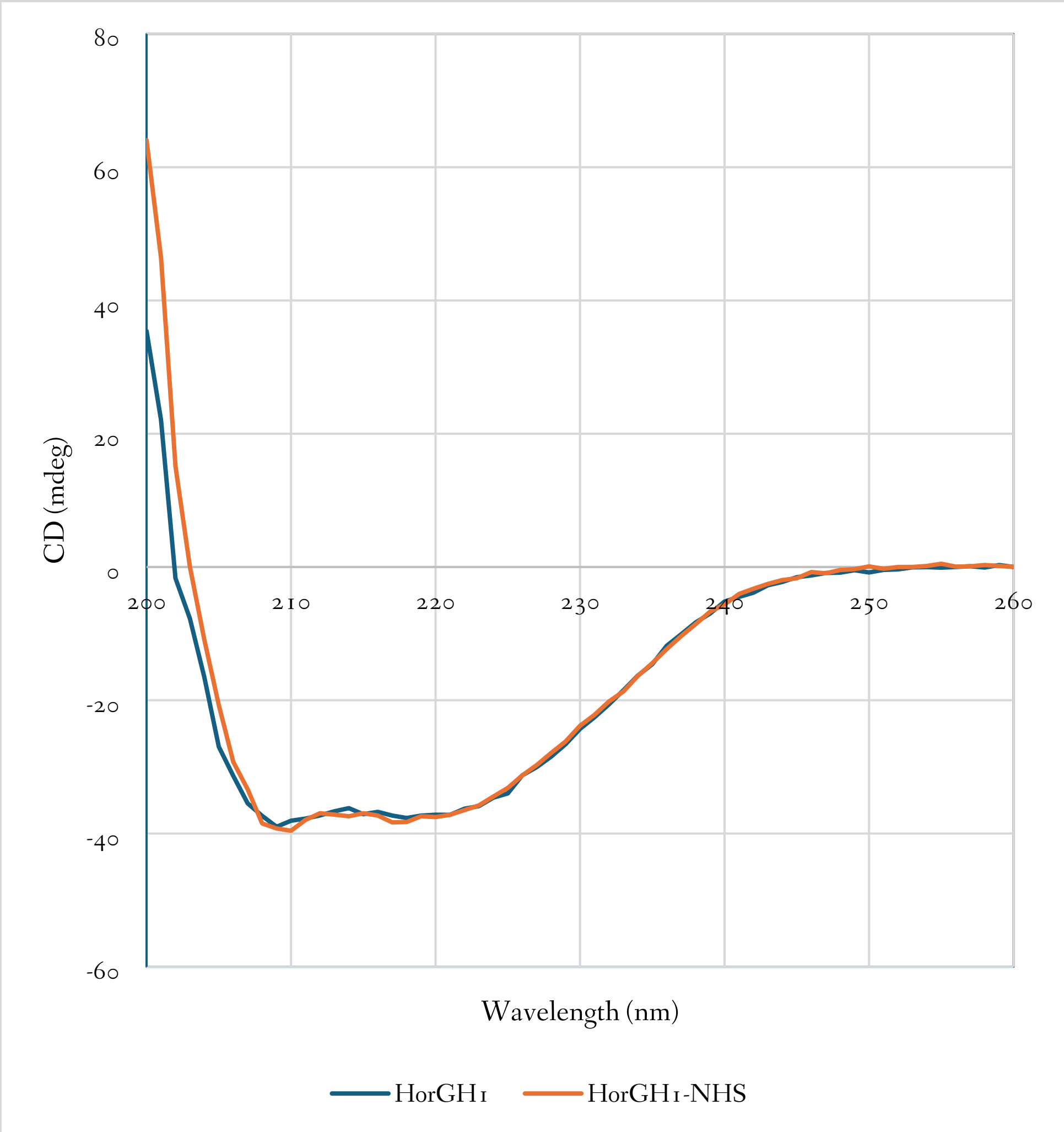

Figure S11: CD spectra showing almost complete overlap between unmodified HorGH1 (blue) and modified with the NHS-containing linker (orange). Protein concentration in both cases was 0.65 mg/mL, in 10 mM KPi buffer pH 7.5.

Immobilised HorGH1 on NHS activated, and control (blocked) supports

a)

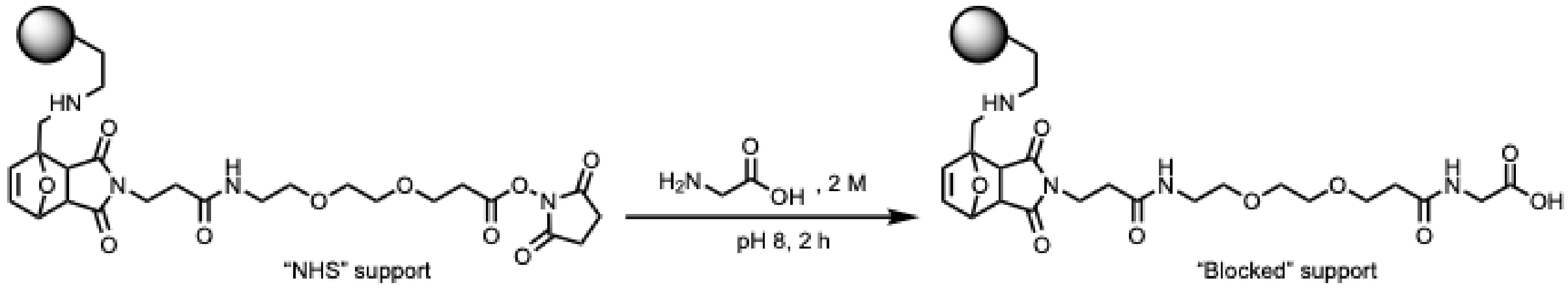

b)

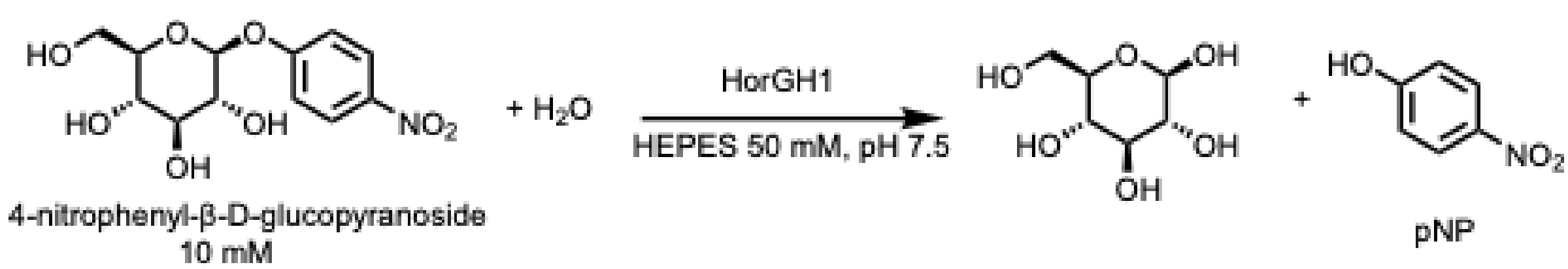

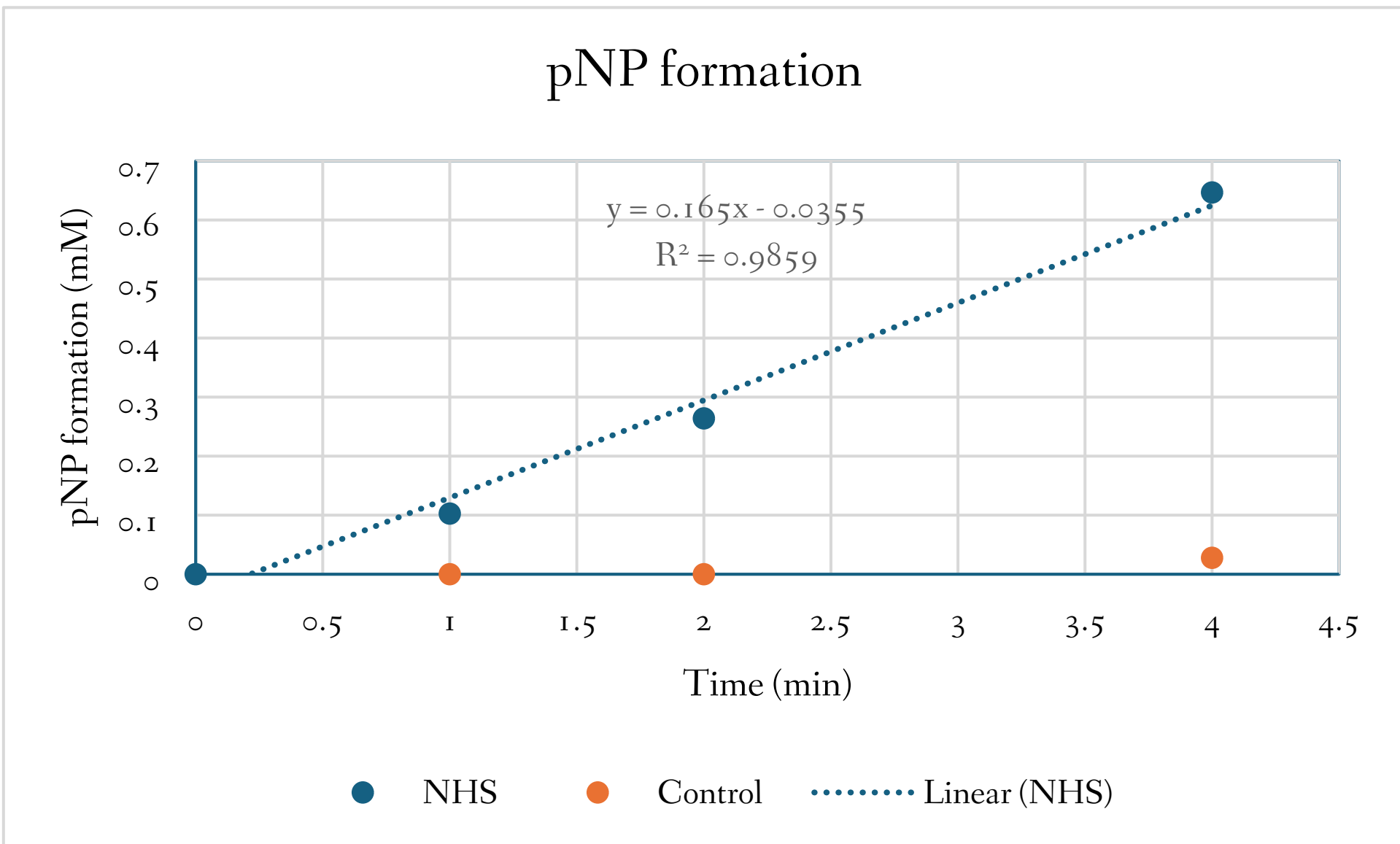

Figure S12: a) Formation of inert support by reacting it with Glycine before introducing the HorGH1 enzyme (blocked). b) Comparison between activated (NHS, blue), and blocked (orange, control) supports for the immobilisation of HorGH1. It is evident that the blocked support was inactive. For the NHS support the following parameters were associated with HorGH1 immobilisation: HorGH1 specific activity: 8 U/mg. Protein offered: 2 $mg_{enzyme}/g_{support}$. IY: 100%. Immobilised Activity: 8.35 U/g. Recovered Activity: 60%.

References

1 D. Roura Padrosa, V. Marchini and F. Paradisi, *Bioinformatics*, 2021, **37**, 2761–2762.

2 https://proteinsuite.inseit.ch/, accessed on 05/04/2026.

3 Q. Guo, L. Gakhar, K. Wickersham, K. Francis, A. Vardi-Kilshtain, D. T. Major, C. M. Cheatum and A. Kohen, *Biochemistry*, 2016, **55**, 2760–2771.

4 N. Hassan, T.-H. Nguyen, M. Intanon, L. D. Kori, B. K. C. Patel, D. Haltrich, C. Divne and T. C. Tan, *Appl. Microbiol. Biotechnol.*, 2015, **99**, 1731–1744.

5 E. Broumidis and F. Paradisi, *Mater. Adv.*, 2025, **6**, 7355–7364.

6 K. A. Majorek, P. J. Porebski, A. Dayal, M. D. Zimmerman, K. Jablonska, A. J. Stewart, M. Chruszcz and W. Minor, *Mol. Immunol.*, 2012, **52**, 174–182.

7 L. Pillet, D. Lim, N. Almulhim, A. I. Benítez-Mateos and F. Paradisi, *Chem. Commun.*, 2022, **58**, 12118–12121.

8 A. C. Stauß, C. Fuchs, P. Jansen, S. Repert, K. Alcock, S. Ludewig and W. Rozhon, *Molecules*, 2024, **29**(14), 3262.

9 B. D. Mather, K. Viswanathan, K. M. Miller and T. E. Long, *Prog. Polym. Sci.*, 2006, **31**, 487–531.

10 S. Mädler, C. Bich, D. Touboul and R. Zenobi, *J. Mass Spectrom.*, 2009, **44**, 694–706.
